# Radiofrequency electrical conductivity reveals a distinct dimension of early human brain development

**DOI:** 10.1038/s41598-026-61290-3

**Published:** 2026-07-14

**Authors:** Arnaud Boutillon, Anthony Price, Roy Kurtzbard, Oliver Gale-Grant, Emil Galanides, Andrew Lawrence, Jeffrey Hand, Dafnis Batalle, Jonathan O’Muircheartaigh, Grainne McAlonan, Joseph V Hajnal, A. David Edwards, Tomoki Arichi, Maria Deprez, Shaihan J Malik

**Affiliations:** 1 Imaging Physics and Engineering Research Department, School of Biomedical Engineering and Imaging Sciences, King’s College London, London, UK; 2 Biomedical Computing Research Department, School of Biomedical Engineering and Imaging Sciences, King’s College London, London, UK; 3 Medical Physics and Clinical Engineering, Guys and St Thomas’ NHS Foundation Trust, London, UK; 4 Department of Forensic and Neurodevelopmental Sciences, Institute of Psychiatry, Psychology, and Neuroscience, King’s College London, London, UK; 5 Early Life Imaging Research Department, School of Biomedical Engineering and Imaging Sciences, King’s College London, London, UK; 6 Department of Paediatric Neurosciences, Evelina London Children’s Hospital, London, UK

**Keywords:** Developmental neuroimaging, Electrical conductivity mapping, Quantitative MRI, Biological techniques, Biophysics, Engineering, Medical research, Neurology, Neuroscience, Physics, Physiology

## Abstract

Quantitative MRI has characterised early human brain development primarily through measures of water mobility, leaving other biophysical properties of maturing tissue unexplored. Here we show that radiofrequency electrical conductivity, reflecting ionic composition, membrane density and extracellular geometry in addition to water content, can be extracted retrospectively and at scale from the phase of conventional MRI acquisitions, revealing a distinct dimension of early brain tissue maturation and state. Applying electrical property tomography to 888 neonatal MRI sessions (784 subjects, 26–45 weeks post-menstrual age) alongside infant and childhood data, we find that whole-brain conductivity declines steeply during the perinatal period and remains independently associated with age after adjustment for mean diffusivity and ventricular volume, confirming that it represents a distinct developmental signal that is not reducible to tissue water content alone. Preterm birth was associated with elevated conductivity at term-equivalent age, particularly in deep grey matter. In neonates with hypoxic–ischaemic encephalopathy (*n* = 25), conductivity was elevated while mean diffusivity was reduced within the same tissue compartments, a dissociation indicating that these measures capture mechanistically distinct aspects of the tissue response to injury. These findings establish radiofrequency electrical properties as a new biophysical window on perinatal brain maturation and its perturbation by injury, extending quantitative MRI beyond measures of water mobility.

**Supplementary Information:**

The online version contains supplementary material available at https://doi.org/10.1038/s41598-026-61290-3.

## Introduction

Early human brain development is characterised by rapid and large-scale change in tissue composition and architecture. During the perinatal period, the brain undergoes substantial reorganisation of its cortical and subcortical structure through fundamental developmental processes including cellular migration and differentiation, synaptogenesis, dendritic arborisation and early myelination^1–4^. These dramatic changes in tissue maturation are accompanied by marked reductions in total water content and progressive restriction of the extracellular space^4–6^. Quantitative MRI can characterise many of these processes, having demonstrated steep developmental trajectories in cortical folding, white matter organisation and subcortical growth during this period^5,7–9^. Diffusion MRI in particular is widely applied as it provides sensitive markers of neural tissue microstructure that reflect underlying cellular organisation and hydration^4,10,11^. However, diffusion measures primarily reflect water molecule motion over millisecond timescales and therefore capture only part of the biophysical changes that accompany brain maturation.

At the radiofrequencies used in clinical MRI (128 MHz at 3T), tissue electrical properties are better described by Maxwell mixture theory than by low-frequency membrane-resistance models, thus both intracellular and extracellular compartments contribute to bulk conductivity, rather than the extracellular space alone^12–17^. Within this framework, conductivity increases with free ion concentration and water content, decreases marginally with increasing membrane interface density owing to residual β/δ-dispersion contributions in highly cellular or myelin-rich tissue, and scales with extracellular space volume fraction as a mixing weight between compartmental conductivities.

Therefore, unlike diffusion MRI, radiofrequency conductivity integrates contributions from ionic and membrane-mediated processes operating at megahertz frequencies—a biophysical regime that is sensitive to different aspects of tissue organisation^12,16^. Experimental studies in developing animal brains demonstrate that conductivity is higher in immature tissue and declines with maturation, consistent with progressive increases in membrane density and cellular complexity during maturation^18–21^.

Electrical conductivity can be estimated non-invasively from conventional MRI phase data using electrical property tomography (EPT), without additional hardware or dedicated pulse sequences^17,22–24^. Specifically, EPT reconstructs electrical properties from the spatial distributions of the radiofrequency magnetic field generated during an MRI scan. In adult brain imaging, EPT has revealed conductivity differences associated with tumour^25^, glioma grade^26^, and ageing^27^, demonstrating that macroscopic electrical properties carry biologically and clinically meaningful information beyond that available from conventional MRI contrasts^28^. However, application of EPT to early human brain development has been limited, and in vivo developmental trajectories of conductivity remain largely unexplored. The perinatal period presents a uniquely favourable context for conductivity imaging: tissue composition changes more rapidly and over a wider dynamic range than at any other stage of the human lifespan offering more contrast^4,6^.

Here, we quantify radiofrequency electrical conductivity across early human brain development, spanning the neonatal period through childhood (26 weeks post-menstrual age to 18 years), using phase-based EPT applied retrospectively to a large developmental MRI dataset. We characterise the perinatal trajectory of conductivity, examine its relationship to mean diffusivity, which shares a dependence on tissue water content, and to ventricular volume, a potential confound due to the high conductivity of cerebrospinal fluid^13–15^. We test the sensitivity of conductivity to common perinatal brain pathologies associated with adverse neurodevelopment, namely preterm birth, which disrupts the normal sequence of perinatal maturation and provides an opportunity to examine how altered developmental trajectories manifest in tissue electrical properties^7,29,30^; and to hypoxic–ischaemic encephalopathy, an acute injury with well-characterised consequences including cytotoxic oedema and ionic dysregulation that are expected to affect radiofrequency conductivity^31–33^. We demonstrate that conductivity exhibits a pronounced developmental trajectory and captures aspects of early brain tissue state and injury that are not described by diffusion MRI, establishing electrical properties as a complementary dimension of human brain development in vivo.

## Results

### Electrical conductivity declines across early human development

**Fig. 1:**
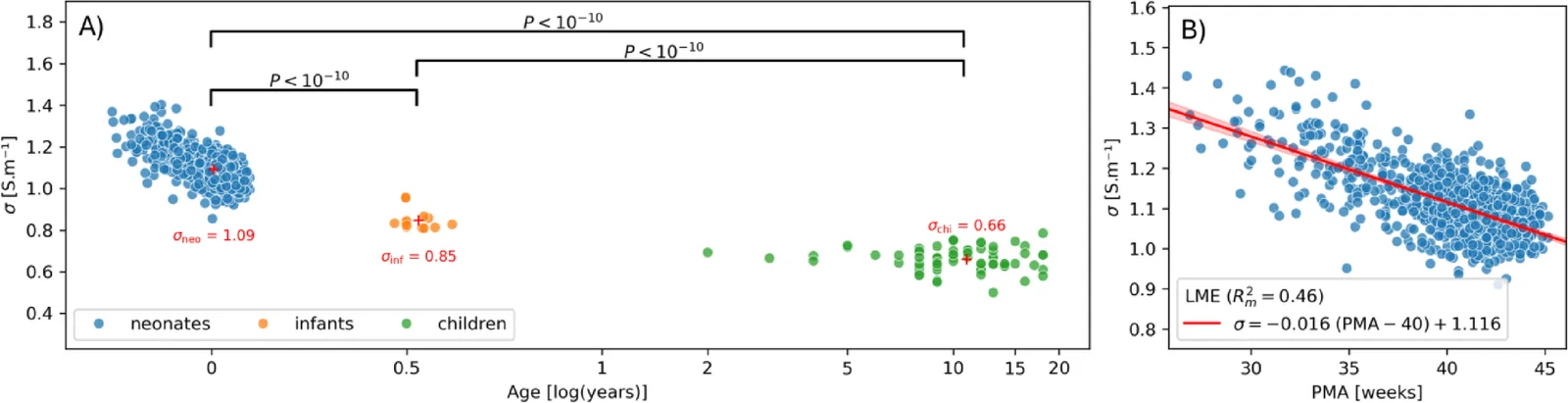
Brain electrical conductivity (σ) across neonatal, infant and child cohorts. (**A**) Scatter plot of σ GRE-derived estimates in neonates (26–45 weeks post-menstrual age – PMA, *n* = 868), infants (~ 6 months old, *n* = 13) and children (2–18 years old, *n* = 77) versus age. The x-axis is linear up to one year, then logarithmic. Age 0 is defined as 40 weeks PMA. Group means were compared using a linear mixed-effects (LME) model and Bonferroni correction was applied for multiple comparisons. Group means are reported for each cohort. (**B**) Linear mixed-effects regression of GRE-derived neonatal σ (*n* = 868) against PMA. Regression was performed using a LME model. The shaded region indicates the 95% confidence interval of the fitted line. The marginal coefficient of determination (${\mathrm{R}}_{\mathrm{m}}^{2}$) is indicated in the panel legend.

Whole-brain parenchymal electrical conductivity (excluding ventricular CSF) differed significantly and systematically across developmental cohorts (group mean differences *P* < 10^− 10^, Bonferroni corrected), with the highest values observed in neonates (σ_neo_ = 1.09 S m^−1^), intermediate values in infants (σ_inf_ = 0.85 S m^−1^), and lower values in children (σ_chi_ = 0.66 S m^−1^; Fig. 1A), consistent with a progressive decline in conductivity across early life. Noting that absolute conductivity estimates are influenced by coil configuration and spatial resolution, which differed across cohorts, the following cross-cohort comparison should be interpreted as indicative of the developmental trend rather than a precise quantitative characterisation.

Within the neonatal cohort specifically (26–45 weeks post-menstrual age - PMA, *n* = 888 sessions), conductivity declined steeply with increasing PMA (Fig. 1B), with a rate of -0.016 S m^−1^ week^−1^ (95% CI [− 0.017, − 0.015], *P* < 10^− 10^, ${\mathrm{R}}_{\mathrm{m}}^{2}$ = 0.46).

### Perinatal conductivity decline is consistent across independent reconstructions

**Fig. 2:**
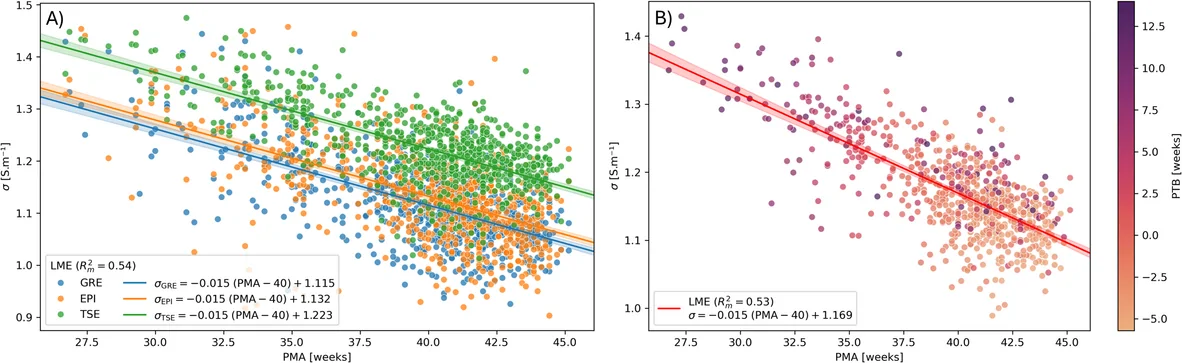
Consistency among GRE, EPI and TSE brain conductivity (σ) reconstructions and their relationship to neonatal development (*n* = 725 sessions with all three reconstructions available). (**A**) Linear mixed-effects (LME) model fitted jointly to GRE, EPI and TSE σ data with post-menstrual age (PMA) as predictor, including reconstruction-type intercepts. Shaded regions indicate 95% confidence intervals of the fitted lines. (**B**) LME regression of weighted average brain σ versus PMA. Point colour indicates degree of prematurity (PTB = 37 − GA [weeks]); positive values indicate preterm birth and negative values indicate post-term birth. The shaded region indicates the 95% confidence interval of the fitted line. The marginal coefficient of determination (${\mathrm{R}}_{\mathrm{m}}^{2}$) is reported in the panel legend for each model.

Conductivity was reconstructed using three different MRI sequences in the neonatal cohort: GRE, EPI and TSE (Material and methods, Conductivity reconstruction). Developmental slopes were concordant across GRE, EPI and TSE reconstructions (GRE = EPI = − 0.015, TSE = − 0.013 S m^−1^ week^−1^; *n* = 725, Supplementary Table S3), with a common slope model providing equivalent fit to the full interaction model (${\mathrm{R}}_{\mathrm{m}}^{2}$ = 0.54 for both; Fig. 2A). A weighted average conductivity estimate was therefore computed for all subsequent analyses. The marginal coefficient of determination for this model was 0.53, indicating that more than half of the variation in σ is explained by PMA during the neonatal period (Fig. 2B).

These findings indicate that the rapid perinatal decrease in conductivity is robust across independent reconstruction strategies and is unlikely to reflect methodological artefact.

### Conductivity decline is not fully attributable to water-related changes

**Fig. 3:**
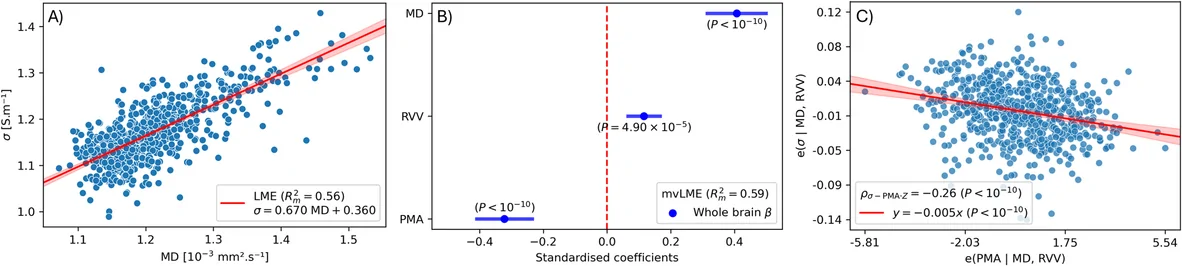
Association between neonatal brain conductivity (σ), mean diffusivity (MD), relative ventricular volume (RVV) and post-menstrual age (PMA; *n* = 662). (**A**) Marginal association between σ and MD from a univariate linear mixed-effects (LME) model, with the shaded region indicating the 95% confidence interval of the fitted line. (**B**) Standardised coefficients (β) from a multivariate linear mixed-effects model (mvLME), with the marginal coefficient of determination (${\mathrm{R}}_{\mathrm{m}}^{2}$) indicated. Coefficients are reported alongside their 95% confidence intervals and exact *P*-values. (**C**) Partial regression plot of σ against PMA after removing shared variance with MD and RVV. The partial correlation coefficient is computed from the residuals *e*(σ | **Z**) and *e*(PMA | **Z**) and reported with its associated significance level. The shaded region indicates the 95% confidence interval of the fitted line. **Z** = {MD, RVV} represents the vector of controlling variables.

Whole-brain conductivity was strongly associated with mean diffusivity (${\mathrm{R}}_{\mathrm{m}}^{2}$ = 0.56; Fig. 3A), consistent with the shared dependence of both measures on tissue water content. In a multivariate linear mixed-effects model incorporating mean diffusivity (MD), relative ventricle volume (RVV) and PMA (*n* = 662, ${\mathrm{R}}_{\mathrm{m}}^{2}$ = 0.59), MD showed the largest standardised association with conductivity (β_MD_ = 0.41, 95% CI [0.31, 0.51], *P* < 10^−10^), followed by PMA (β_PMA_ = − 0.32, 95% CI [− 0.41, − 0.23], *P* < 10^− 10^) and RVV (β_RVV_ = 0.12, 95% CI [0.06, 0.17], *P* = 4.90 × 10^−5^; Fig. 3B). PMA retained a significant independent association with conductivity after mutual adjustment; the partial correlation was substantially attenuated relative to its marginal value, confirming shared variance with other predictors while demonstrating an independent contribution (Fig. 3C).

**Fig. 4:**
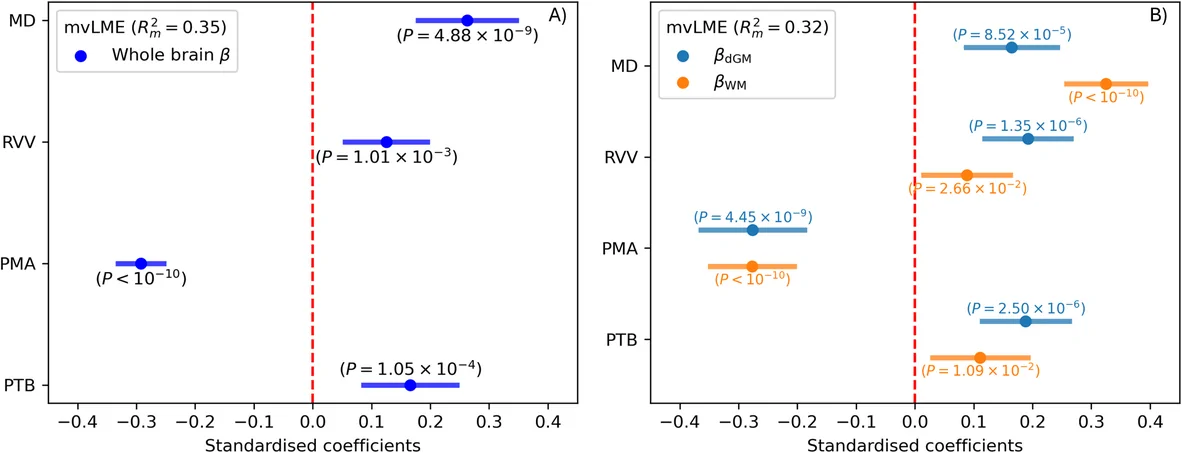
Association between neonatal conductivity (σ), mean diffusivity (MD), relative ventricular volume (RVV), preterm birth (PTB) and post-menstrual age (PMA; *n* = 534). (**A**) Whole-brain standardised coefficients (β) from a multivariate linear mixed-effects model (mvLME). (**B**) Deep grey matter (dGM) and white matter (WM) standardised coefficients from a mvLME. For each model, the marginal coefficient of determination (${\mathrm{R}}_{\mathrm{m}}^{2}$) is indicated. Coefficients are reported alongside their 95% confidence intervals and exact *P*-values.

The persistence of a significant independent PMA association after adjustment for MD indicates that the developmental conductivity decline is a distinct effect that is not solely explained by developmental changes in water content and diffusion properties. Furthermore, the positive RVV association, modest in magnitude, is consistent with partial volume contamination from ventricular cerebrospinal fluid (CSF) rather than a primary biological effect and thus does not account for the principal developmental trajectory.

### Prematurity is associated with elevated conductivity at term-equivalent age

In neonates born preterm, conductivity at any given post-menstrual age was significantly higher, with a clear relationship between the degree of prematurity and conductivity visible across the perinatal interval (Fig. 2B). In a whole-brain mixed-effects model adjusting for MD, RVV and PMA (*n* = 534, ${\mathrm{R}}_{\mathrm{m}}^{2}$ = 0.35), greater degree of prematurity (PTB, defined as weeks before 37 weeks GA) was independently associated with higher conductivity (β_PTB_ = 0.17, 95% CI [0.08, 0.25], *P* = 1.05 × 10^−4^; Fig. 4A), indicating that the prematurity effect is not fully explained by concurrent differences in water content or ventricular volume.

Tissue-level modelling revealed positive associations between prematurity and conductivity in both deep grey matter (${\beta}_{\mathrm{PTB}}^{\mathrm{dGM}}$ = 0.19, 95% CI [0.11, 0.27], *P* = 2.50 × 10^− 6^) and white matter (${\beta}_{\mathrm{PTB}}^{\mathrm{WM}}$ = 0.11, 95% CI [0.03, 0.20], *P* = 0.011; Fig. 4B), after adjustment for tissue-specific MD, RVV and PMA (Fig. 4B). The unstandardised Tissue×PTB interaction term was statistically significant (*P* = 0.002; Supplementary Table S6), confirming that the prematurity effect on conductivity was significantly greater in deep grey matter than white matter.

### HIE is associated with elevated conductivity and dissociation from diffusion measures

**Fig. 5:**
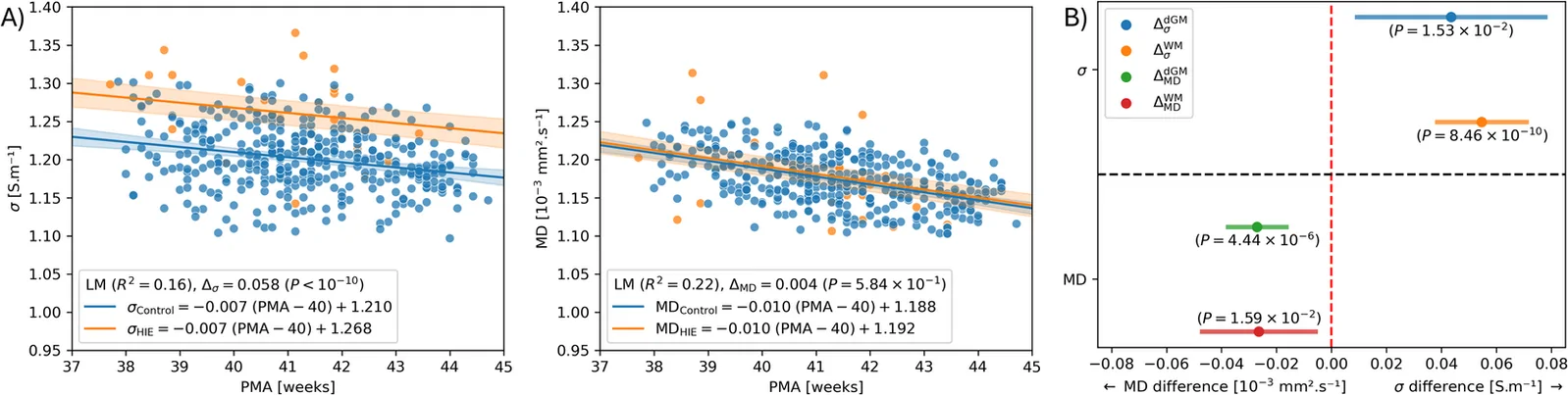
Conductivity (σ) and mean diffusivity (MD) in neonates with HIE (*n* = 25) and term-born controls (*n* = 379). (**A**) Whole-brain σ and MD with respect to post-menstrual age (PMA). For each metric, a linear model (LM) was fitted to control and HIE data with PMA as predictor variable, with separate intercepts for each group and a common slope. The coefficient of determination R^2^, and group mean difference (Δ) with its 95% confidence interval are reported for each model. (**B**) Tissue-level group differences (Δ) in σ and mean MD after adjustment for PMA. Effects are displayed on a shared axis: rightward displacement indicates higher conductivity values in HIE relative to controls; leftward displacement indicates MD lower values in HIE relative to controls. The dashed horizontal line separates conductivity effects (above) from MD effects (below). Group mean differences are shown with 95% confidence intervals and exact *P*-values.

In models adjusting for PMA, neonates with hypoxic–ischaemic encephalopathy (HIE) demonstrated significantly elevated whole-brain conductivity relative to term-born controls (Δ_σ_ = 0.058 S m^−1^, 95% CI [0.041, 0.075], *P* < 10^−10^; Fig. 5A), corresponding to a 4.8% increase relative to the control mean. In contrast, whole-brain MD did not differ significantly between groups (*P* = 0.584), despite the two measures being strongly correlated in the healthy developmental cohort (Fig. 3A). This dissociation between conductivity and diffusion was replicated at the tissue level: conductivity was elevated in both deep grey matter (${\Delta}_{\upsigma}^{\mathrm{dGM}}$= 0.043 S m^−1^, 95% CI [0.008, 0.078], *P* = 0.015) and white matter (${\Delta}_{\upsigma}^{\mathrm{WM}}$ = 0.055 S m^−1^, 95% CI [0.038, 0.072], *P* = 8.46 × 10^− 10^), while MD was reduced in the same tissues (${\Delta}_{\mathrm{MD}}^{\mathrm{dGM}}$ = −0.027 × 10^− 3^ mm² s^−1^, 95% CI [ − 0.038, − 0.016], *P* = 4.44 × 10^− 6^; ${\Delta\:}_{\mathrm{MD}}^{\mathrm{WM}}$ = −0.026 × 10^− 3^ mm² s^−1^, 95% CI [− 0.048, − 0.005], *P* = 1.59 × 10^− 2^; Fig. 5B). The simultaneous elevation of conductivity and reduction of MD within the same tissue compartments confirms a genuine dissociation between these two imaging measurements following hypoxic–ischaemic injury.

In a tissue-level multivariate model adjusting for PMA, MD and RVV, HIE remained independently associated with elevated conductivity in both deep grey matter (${\Delta}_{\upsigma}^{\mathrm{dGM}}$ = 0.050 S m^−1^, 95% CI [0.022, 0.078], *P* = 5.44 × 10^− 4^) and white matter (${\Delta}_{\upsigma}^{\mathrm{WM}}$ = 0.062 S m^−1^, 95% CI [0.034, 0.090], *P* = 1.50 × 10^− 5^; Supplementary Table S7), confirming that the conductivity elevation is not attributable to group differences in water content or ventricular volume.

An exploratory multivariate logistic regression model, fitted to predict HIE status from σ_dGM_, σ_WM_, MD_dGM_, MD_WM_, RVV and PMA, demonstrated that tissue-level conductivity contributed independently to HIE classification after adjustment for diffusion measures (full model output in Supplementary Fig. S7). Given the small HIE sample size (*n* = 25) within which there was probable clinical heterogeneity and absence of outcome data, these results should be considered hypothesis-generating only and are not intended to support clinical diagnostic utility.

## Discussion

Radiofrequency electrical conductivity of tissue can be measured with MRI and reflects tissue composition rather than neuronal electrical activity. Using existing MRI data to estimate conductivity, we found that conductivity exhibits a pronounced developmental trajectory across early human brain maturation, characterised by a steep perinatal decline that is robust across three independent MRI acquisitions and reconstruction approaches and crucially is not reducible to concurrent changes in water diffusivity. We further demonstrate that the conductivity contrast is sensitive to pathological mechanisms with prematurity associated with elevated conductivity at term-equivalent age, and neonates with hypoxic–ischaemic encephalopathy showing a striking dissociation in which conductivity is elevated while diffusivity is simultaneously reduced in the same tissue compartments. Together, these findings identify electrical conductivity as a distinct dimension of brain tissue state that complements established MRI markers of maturation, capturing aspects of tissue composition and injury that are not reflected by measures of water mobility alone.

### Developmental change in human brain electrical conductivity

The observed trajectory is consistent with established biophysical properties of developing brain tissue. Within the neonatal period alone, conductivity declined at approximately 1.3% per week, with a 40% reduction between the neonatal and childhood cohorts. At radiofrequencies, macroscopic conductivity cumulatively reflects water fraction, ionic composition, extracellular space geometry (mostly characterised by the volume fraction and tortuosity of the extracellular space) and membrane interface density^6,12–16,34^. During the perinatal period, marked developmental changes in cellular size and neuronal and non-neuronal cell differentiation, result in increasing membrane complexity, synaptogenesis and early myelination^1,4,11^. These changes reduce free water availability and extracellular space volume, and may affect other contributing factors, all of which lead to the observed strong decrease in conductivity. Our findings are also in keeping with experimental measurements in developing animal brain which similarly demonstrate higher conductivity in immature tissue, which declines during maturation^18–21^, with the present findings extending these observations to in vivo human development at substantially greater scale.

Childhood conductivity values (${\upsigma}_{\mathrm{Chi}}$ = 0.66 S m^−1^) are consistent with adult EPT measurements reported in the literature (${\upsigma}_{\mathrm{GM}}$ = 0.76 S m^-1^ and ${\upsigma}_{\mathrm{W}\mathrm{M}}$ = 0.53 S m^−1^)^24^, supporting the plausibility of the estimates. The apparent stabilisation of conductivity after approximately two years of age (Fig. 1A) is also consistent with prior dosimetry studies demonstrating stable dielectric properties beyond early childhood^35,36^. Our results are also consistent with a recent study showing age-dependent conductivity across the lifespan using water-content-based EPT^37^. In that approach, conductivity is assumed to be a monotonic function of tissue water content, and thus intrinsically cannot capture contributions from ionic composition or membrane organisation. The persistence of an independent age association after adjustment for mean diffusivity in the present data, and the divergence of conductivity and diffusion following hypoxic–ischaemic injury, are consistent with such non-water contributions to the developmental conductivity signal.

### Relationship between conductivity and diffusion-derived measures

Whole-brain conductivity was strongly associated with mean diffusivity (Fig. 3A), consistent with the shared dependence of both measures on tissue water content during development^4,11^. Mean diffusivity was therefore used as a proxy for tissue water content in subsequent multivariate analyses^5,40^, in the absence of direct quantitative water content measurements in this cohort. Crucially however, post-menstrual age retained an independent association with conductivity even after adjustment for MD and ventricular volume (Fig. 3B, C). This dissociation is biophysically interpretable. Diffusion MRI reflects microscopic water displacement over millisecond timescales and is primarily sensitive to tissue compartmentalisation. In contrast, radiofrequency conductivity at 128 MHz reflects bulk dielectric behaviour determined by ionic composition, extracellular space geometry and membrane polarisation at megahertz frequencies, a regime in which the influence of membrane microstructure differs from that governing low-frequency or direct-current conduction^12,16^. The residual age-dependent component after adjustment for diffusivity is therefore consistent with developmental changes in tissue ionic environment and membrane organisation that extend beyond water mobility alone.

### Prematurity and altered developmental trajectories of electrical conductivity

Prematurity was independently associated with elevated conductivity at term-equivalent age at both whole-brain (β_PTB_ = 0.17; Fig. 4A) and tissue levels, with a stronger effect in deep grey matter (β_PTB_ = 0.19) than white matter (β_PTB_ = 0.11; Fig. 4B), a difference confirmed by a significant Tissue×PTB interaction (*P* = 0.002), remaining significant after adjustment for mean diffusivity and ventricular volume.

The persistence of this effect after adjustment for mean diffusivity suggests that prematurity is associated with differences in tissue composition that are not fully captured by water diffusivity alone. Elevated conductivity in this context is consistent with an altered and likely less mature tissue microenvironment characterised by higher residual water content, greater extracellular volume and lower membrane density than expected for a given post-menstrual age. Preterm birth disrupts the normal sequence of perinatal maturation, with consequences including delayed pre-oligodendrocyte differentiation, reduced dendritic arborisation and altered microstructural organisation that have been demonstrated using advanced diffusion model at term-equivalent age^7,10,29,30,38^. These processes alter the cellular and extracellular environment in ways that would be expected to affect radiofrequency conductivity through changes in membrane density and extracellular geometry, complementing the water-mobility signal captured by diffusion imaging. Deep grey matter structures, including the thalamus and basal ganglia, undergo particularly rapid maturation during the perinatal period and are recognised as especially vulnerable to prematurity-related disruption^7,39^, consistent with the stronger prematurity effect observed in this compartment.

### Divergence of conductivity and diffusion measures following hypoxic–ischaemic injury

Neonates with hypoxic–ischaemic encephalopathy demonstrated significantly elevated whole-brain conductivity relative to term-born controls (4.8% increase), while whole-brain mean diffusivity did not differ (Fig. 5A). This dissociation was replicated at the tissue level, where conductivity was elevated and mean diffusivity simultaneously reduced in both deep grey matter and white matter (Fig. 5B), consistent with observations from an animal model of hypoxic–ischaemic injury in which conductivity increased while diffusivity decreased following insult^40^. This dissociation likely reflects distinct pathophysiological processes that unfold across the neonatal period following hypoxic–ischaemic injury. Cytotoxic oedema restricts intracellular water motion and reduces diffusivity, a well-established pattern in neonatal HIE^33,41–43^. All neonates with HIE were imaged between 4 and 10 days postnatal age, a window corresponding to the secondary–tertiary phases of injury during which a range of processes including alterations in cellular metabolism, extracellular metabolite concentrations, ion transporter function and pH regulation are known to occur^31–33^. To fully unpick these factors and their specific contributions to radiofrequency conductivity, longitudinal, multi-modal investigation is required. Nonetheless, the conductivity elevation persisted after adjustment for mean diffusivity and ventricular volume in tissue-level multivariate models (Supplementary Table S7), confirming that conductivity and diffusion capture distinct aspects of the pathophysiological response to hypoxic–ischaemic injury.

### Methodological considerations

EPT methods require spatial differentiation of the complex MRI data, which amplifies noise, and the source acquisitions in this study were not optimised for conductivity reconstruction (Supplementary Table S2). Analyses therefore focused on whole-brain and tissue-compartment averages rather than voxelwise inference, with ROI erosion applied to mitigate boundary artefacts that arise at tissue interfaces due to the finite spatial kernel used in the differentiation step^44^. Despite these challenges, developmental trajectories were concordant across GRE, EPI and TSE reconstructions, three fully independent MRI acquisitions, each reconstructed using different mathematical frameworks (homogeneous Helmholtz for GRE, heterogeneous Helmholtz for EPI and TSE; Supplementary Fig. S1). This convergence across independent data and methods provides strong evidence that the observed perinatal decline reflects a genuine tissue property rather than a methodological artefact of any single acquisition or reconstruction approach. For the HIE cohort, only TSE data were available; however, the strong agreement between methods in the larger developmental dataset suggests that reconstruction-specific bias is unlikely to account for the observed group differences.

A further limitation concerns methodological differences across cohorts that may influence absolute conductivity estimates. Neonatal, infant and child data had to be unavoidably acquired using different head coils, spatial resolutions and EPT input sequences due to age-specific differences in head size and safety limits (Supplementary Table S2). Coil geometry will impact B_1_ field sensitivity and EPT reconstruction quality, while spatial resolution differences increase partial volume contamination at lower resolution. Cross-cohort comparisons of absolute conductivity values should therefore be interpreted with caution, and the full lifespan trajectory (Fig. 1A) considered indicative rather than a precise quantitative characterisation. Nonetheless, conductivity values in the child cohort are consistent with both the adult literature^24^ and recent water-content-based EPT measurements across the lifespan^37^. The apparent stabilisation beyond early childhood should be interpreted with caution pending replication. Importantly, consistent acquisition parameters were used for the within-cohort findings, including the key characterization of the neonatal developmental trajectory, prematurity effect and HIE group comparison, and thus are not affected by these cross-cohort differences.

Ventricular cerebrospinal fluid exhibits substantially higher conductivity than brain parenchyma at radiofrequencies^13–15^, and ventricular size varies across the preterm-to-term range. Although ventricles were excluded from parenchymal conductivity calculations, the spatial smoothing required by EPT reconstruction can propagate high CSF conductivity values into adjacent tissue voxels^44^, potentially inflating parenchymal estimates in subjects with larger ventricles. Relative ventricular volume was therefore included as a covariate in all multivariate models. Although RVV was independently associated with conductivity, its inclusion did not attenuate the developmental association with post-menstrual age, indicating that the observed trajectory is not attributable to partial volume contamination from CSF.

### Limitations and future directions

This study has several limitations. The cross-sectional design precludes direct inference on within-individual developmental trajectories, and longitudinal data will be required to characterise temporal evolution beyond early childhood. The HIE cohort was relatively small (*n* = 25), heterogeneous with respect to injury pattern and severity, the timing of imaging after the initial injury, clinical management, and lacked long-term neurodevelopmental outcome data. Consequently, it is not possible to determine whether the observed conductivity elevation identifies neonates at risk of adverse outcome or simply reflects acute injury state at the time of imaging, nor whether the effect scales with injury severity, as suggested by animal model data^40^. The absence of direct measures of tissue water content, ionic composition and membrane density in our sample further constrains mechanistic interpretation. Our HIE findings should therefore not be interpreted as evidence of clinical diagnostic utility, and the observed conductivity–diffusion dissociation warrants prospective investigation in larger severity-stratified and outcome-linked cohorts.

Future multimodal studies combining EPT with quantitative estimation of water content and other measures such as sodium MRI may enable separation of hydration and ionic contributions to the conductivity signal, providing greater mechanistic insight than is possible with EPT alone. It is worth noting that ‘water content’ is not a single well-defined quantity: classical dielectric studies define it gravimetrically from dried ex vivo tissue^12^, recent in vivo approaches infer it from T1 relaxation^37^, and here we use MD as a surrogate. Each reflects a different aspect of tissue water, and the relationship between these measures and conductivity is an open question that deserves dedicated investigation. Because phase-based EPT can be applied retrospectively to conventional MRI phase data, this approach is in principle immediately scalable to large existing developmental and clinical datasets. A practical caveat is that although phase information is inherent to every MRI acquisition, it is routinely discarded during image reconstruction. The present study was possible because the dHCP study archived complex-valued raw data. Broader adoption of retrospective conductivity mapping will require preserving phase data as standard archiving practice^23,25,26^.

## Concluding statement

Human brain electrical conductivity declines steeply during the perinatal period, is not reducible to concurrent changes in water diffusivity, and is specifically altered by perinatal brain pathology. This information, already encoded in the phase of conventional MRI acquisitions, establishes electrical properties as a new biophysical dimension of early brain development that complements existing measures of water mobility. We hypothesise that several overlapping processes occurring during the perinatal period, such as the onset of myelinations, synaptogenesis, and dendritic arborisation, which dramatically increase membrane surface area, and the contraction of the extracellular space as cells proliferate and enlarge, act independently of water fraction reduction to reduce bulk conductivity.

## Materials and methods

### Participants and cohorts

Participants were drawn from four cohorts, all scanned at a single site on a 3T Philips Achieva MRI system (Larmor frequency 128 MHz). A summary of cohort characteristics and imaging parameters is provided in Supplementary Tables S1 and S2.

#### Developing human connectome project (dHCP)

Neonatal data were obtained from the developing Human Connectome Project (dHCP), a large-scale study of early human brain development^45,46^. The cohort comprised 784 neonates (206 preterm, 578 term) born between 23 and 43 weeks gestational age (GA) and scanned between 26 and 45 weeks post-menstrual age (PMA; mean 40.0 ± 3.4 weeks) across 888 sessions. Diffusion-weighted imaging was available in 773 sessions.

#### Brain injury in hypoxic–ischaemic encephalopathy cohort (BI-HIE)

Thirty-five neonates with a clinical diagnosis of hypoxic–ischaemic encephalopathy (HIE) were scanned prospectively at term-equivalent age (37–47 weeks PMA; mean 40.8 ± 2.1 weeks). To reduce variability in injury stage, analyses were restricted to term-born neonates imaged at 4–10 days postnatal age, yielding *n* = 25 HIE cases corresponding to the secondary and tertiary phases of HIE. These were compared with *n* = 379 term-born dHCP neonates scanned at comparable PMA, serving as controls.

#### Brain imaging in babies (BIBS)

Infant data were drawn from the BIBS study, a longitudinal investigation of early postnatal brain development. A subset of participants was enrolled in both the BIBS and dHCP studies under dual consent. Fourteen sessions from 11 infants aged 5.5–7.5 months were available; 13 passed quality control for conductivity analysis.

#### Research in imaging of childhood epilepsy (RICE)

Child data were drawn from the RICE study^47,48^. Seventy-eight sessions from 58 children aged 2–18 years (mean 10.8 ± 3.9 years) were available; 77 passed quality control. Conductivity did not differ significantly between epilepsy and non-epilepsy subgroups (*P* = 0.55*)*, supporting inclusion of the full cohort in developmental analyses.

### Imaging protocol and MRI acquisition

Neonatal imaging used a dedicated neonatal system with a 32-channel phased-array infant head coil; infant and child imaging used a 32-channel adult head coil. All neonates were scanned during natural sleep without sedation. All studies received institutional ethics committee approval and written parental consent was obtained from all participants.

For dHCP neonates, imaging protocols have been described previously^45,46^. Conductivity estimation was derived from three conventional acquisitions: multi-coil gradient echo (GRE) sensitivity reference scans, spin-echo echo planar imaging (EPI), and T2-weighted turbo spin-echo (TSE). Diffusion-weighted imaging used single-shot spin-echo EPI with multiple b-values optimised for neonatal imaging^49^. Acquisition parameters varied by sequence and cohort and are detailed in Supplementary Table S2.

### Conductivity reconstruction

Electrical conductivity (σ) was reconstructed using phase-based electrical property tomography. GRE reconstructions used the single-acquisition electrical properties (SAEP) method based on the homogeneous Helmholtz equation^22,23^ EPI and TSE reconstructions used phase-only conductivity reconstruction (POCR) based on the heterogeneous Helmholtz equation^24^ Preprocessing steps included Gibbs ringing correction, B0 distortion correction for EPI, flow void artefact interpolation, and Gaussian smoothing^50,51^. Spatial derivatives were computed using finite difference schemes. Full implementation details are provided in Supplementary Methods S3.

From the *n* = 725 dHCP sessions with all three reconstructions available, developmental slopes were compared across methods using linear mixed-effects modelling. A likelihood ratio test detected a statistically significant but negligible interaction between method and PMA slope (χ²(2) = 7.22, *P* = 0.027; Supplementary Table S3), with overlapping confidence intervals across GRE, EPI and TSE slopes estimates. The marginal coefficient of determination was identical between the full and restricted models (0.54), confirming negligible interaction. Conductivity estimates were therefore aggregated into a weighted average:

$${{\sigma}}_{i}=\frac{\sum_{k}{w}_{ik}{\widehat{\sigma}}_{ik}}{\sum_{k}{w}_{ik}}$$

${w}_{ik}$ were defined as the inverse of the within-brain spatial standard deviation of reconstruction *k* for subject *i*, computed independently for each subject and each available reconstruction. Subjects with noisier reconstructions for a given method therefore receive lower weights for that method, with the weighted average drawn more strongly from higher-quality estimates. This weighting scheme optimises precision by down-weighting noisy reconstructions but does not correct for systematic between-method intercept offsets. Sensitivity analyses confirmed that neither the weighting scheme nor the intercept offsets materially influenced the reported statistical findings. Systematic and proportional biases between methods are characterised in Supplementary Fig. S3.

For the BIBS and RICE cohorts, only GRE-derived conductivity was available. For the HIE cohort, only TSE-derived conductivity was available.

### Image processing and regional measures

Neonatal brain tissue was segmented using the dHCP automated pipeline providing labels for deep grey matter (dGM), white matter (WM), ventricles and CSF^9^. For BIBS and RICE cohorts, whole-brain masks were delineated from body coil magnitude images (Supplementary Methods S1).

For neonates, whole-brain conductivity was computed as the median over all parenchymal voxels, excluding ventricular CSF. Tissue-specific conductivity (σ_dGM_, σ_WM_) and mean diffusivity (MD) were defined as the median within each segmented compartment, using identical masks for both measures. Median values were used in preference to means, as they are more robust to EPT outlier voxels arising from misregistration or reconstruction discontinuities. Relative ventricular volume (RVV) was calculated as ventricular volume divided by total intracranial volume.

Because phase-based EPT reconstruction amplifies noise through spatial differentiation, and because source acquisitions were not optimised for EPT, primary analyses used compartment-averaged rather than voxelwise conductivity measures.

### Statistical analysis

All statistical analyses were performed in Python (v3.10) using the statsmodels library (v0.14.2)^52^. Univariate and multivariate linear mixed-effects (LME and mvLME) models with subject-level random intercepts were used throughout to account for repeated sessions within subjects. Model diagnostics including variance inflation factors, outlier sensitivity and leverage point analyses are reported in Supplementary Methods S4.

#### Cross-cohort comparison

Brain conductivity of neonates (*n* = 868), infants (*n* = 13) and children (*n* = 77) derived from GRE measurements were compared statistically using an LME model of the form:

$$\:\sigma\:\sim\:\mathrm{Cohort}+(1\mid\:\mathrm{Subject})$$

where Cohort is a three-level categorical factor (neonates, infants, children). Multiple comparisons were controlled using Bonferroni correction. GRE-derived conductivity was used as it was the only reconstruction available across all three cohorts. Ventricles and CSF were included in all brain masks, as it was not possible to exclude them in the BIBS and RICE datasets.

For all subsequent neonatal analyses, the dHCP brain tissue labels allowed us to remove the ventricles and CSF from the conductivity calculation.

#### Developmental models

To investigate the relationship between brain $\:\sigma\:$ and neonatal development (i.e. post-menstrual age; PMA), we performed a univariate linear regression using GRE-derived data from the dHCP cohort (*n* = 868), using the LME model:

$$\:\sigma\:\sim\:\mathrm{PMA}+(1\mid\:\mathrm{Subject})$$

Following confirmation of agreement between neonatal GRE, EPI and TSE-derived measurements (*n* = 725 sessions with all three measurements available, Supplementary Table S3), we used the weighted average conductivity measurements for all subsequent analyses.

The primary whole-brain developmental model was:

$$\:\sigma\:\sim\:\mathrm{MD}+\mathrm{RVV}+\mathrm{PMA}+(1\mid\:\mathrm{Subject})$$

MD was included as a covariate to account for the shared variance between conductivity and tissue water content, given the known dependence of both measures on brain hydration during development. RVV was included to control for partial volume contamination from ventricular CSF, whose conductivity substantially exceeds that of brain parenchyma. Pairwise associations between all model variables are shown in Supplementary Fig. S4. A total of *n* = 662 neonatal data points were included, as brain MD was not available in all dHCP sessions.

Prematurity was examined as a modulator of the developmental conductivity trajectory, given that preterm birth disrupts the normal sequence of perinatal maturation and is associated with altered brain development at term-equivalent age. Preterm birth (PTB) was operationalised as a continuous variable to preserve the graded nature of prematurity-related developmental disruption rather than imposing a binary preterm/term distinction. This analysis was restricted to scans at PMA ≥ 37 weeks to ensure that both term-born and preterm neonates were compared at overlapping scan ages (*n* = 534).

PTB was defined as PTB = 37 – GA (positive values indicate preterm birth and negative values indicate post-term birth), and incorporated into an extended model:

$$\:\sigma\:\sim\:\mathrm{MD}+\mathrm{RVV}+\mathrm{PMA}+\mathrm{PTB}+(1\mid\:\mathrm{Subject})$$

Deep grey matter and white matter were selected for tissue-level analyses as the two largest and most reliably segmented parenchymal compartments in the neonatal brain, and because they represent distinct developmental trajectories; dGM undergoes rapid cellular maturation and dendritic growth while WM is characterised by progressive myelination and axonal organisation during the perinatal period. Tissue-level analyses examined dGM and WM simultaneously using a multivariate model with tissue-specific interaction terms:

$$\:\sigma\:\sim\:\mathrm{Tissue}\times\:(\mathrm{MD}+\mathrm{RVV}+\mathrm{PMA}+\mathrm{PTB})+(1\mid\:\mathrm{Subject})$$

where Tissue is a two-level categorical factor (dGM, WM). This specification estimates tissue-specific intercepts and covariate effects within a single model, with the Tissue×PTB interaction term directly testing whether the prematurity effect on conductivity differs between tissue classes.

#### HIE models

HIE was examined as a model of acute neonatal brain injury in which the primary pathophysiological cascade is expected to produce ionic dysregulation and consequent changes in tissue electrical conductivity^17,33^. Conductivity was therefore hypothesised to be elevated in HIE relative to controls, and critically, to diverge from diffusion MRI measures that reflect water mobility rather than ionic homeostasis. HIE group differences were assessed using separate linear models for whole-brain and tissue-level conductivity and MD, adjusting for PMA:

$$\:\sigma\:\sim\:\mathrm{HIE}+\mathrm{PMA}$$

$$\:\mathrm{MD}\sim\:\mathrm{HIE}+\mathrm{PMA}$$

$$\:\sigma\:\sim\:\mathrm{Tissue}\times\:(\mathrm{MD}+\mathrm{RVV}+\mathrm{PMA}+\mathrm{HIE})+(1\mid\:\mathrm{Subject})$$

Group mean differences (Δ) with 95% confidence intervals were derived from model coefficients. The HIE coefficient from each model provides the estimated group difference in conductivity or MD at equivalent PMA after covariate adjustment. A total of *n* = 379 control neonates and *n* = 25 HIE neonates were included.

#### Exploratory HIE classification

A multivariate logistic regression model was fitted to predict HIE status using σ_dGM_, σ_WM_, MD_dGM_, MD_WM_, RVV and PMA as predictors. Given the small HIE sample size (*n* = 25), this analysis is considered exploratory and results should be interpreted with caution. Full model output is provided in Supplementary Fig. S7.

#### Reporting

For all models, fixed effect coefficients are reported alongside 95% confidence intervals and exact *P*-values. Standardised coefficients (β) were computed by refitting models on *z*-score normalised predictors to permit direct comparison of effect magnitudes across variables with different scales. Marginal coefficients of determination (${\mathrm{R}}_{\mathrm{m}}^{2}$) are reported as a measure of variance explained by fixed effects alone. Each model was pre-specified to test a defined hypothesis; no correction for multiple comparisons was applied across models, as each addresses a distinct scientific question.

## Supplementary Information

Below is the link to the electronic supplementary material.

Supplementary Material 1

## Data Availability

The EPT reconstruction algorithms and statistical analysis notebooks are publicly available at https://github.com/aboutill/nano-eptk. We also provide tabulated data containing all extracted EP measurements (σ and ε_r_), along with relevant covariates for each study cohort (dHCP, BI-HIE, BIBS and RICE). In addition, the GRE phantom scans is included. The rest of the data that support the findings of this study are available on reasonable request from the corresponding author.
