# Supplementary material for: Radiofrequency electrical conductivity reveals a distinct dimension of early human brain development
Source: Sci Rep. 2026 Jul 14;16:32188. doi: 10.1038/s41598-026-61290-3 (PMC13661241; doi:10.1038/s41598-026-61290-3)
Supplement: Supplementary file 1 [file 41598_2026_61290_MOESM1_ESM.pdf]

Supplementary Materials

Radiofrequency electrical conductivity reveals a distinct dimension of early human brain development

Arnaud Boutillon, Anthony Price, Roy Kurtzbard, Oliver Gale-Grant, Emil Galanides, Andrew Lawrence, Jeffrey Hand, Dafnis Batalle, Jonathan O'Muircheartaigh, Grainne McAlonan, Joseph Hajnal, A. David Edwards, Tomoki Arichi, Maria Deprez, Shaihan Malik

Supplementary materials and methods

S1. Cohort characteristics

**A) Clinical information**						
Cohort name	Cohort description				Age (mean ± STD [min, max])	
	Type	Subjects	Sessions	Pre / Term	GA	PMA
dHCP	Neonates	784	888	206 / 578	37.9 ± 4.2 [23, 43] weeks	40.0 ± 3.4 [26, 45] weeks
BI-HIE	Neonates	35	35	3 / 32	39.5 ± 1.7 [36, 42] weeks	40.8 ± 2.1 [37, 47] weeks
BIBS	Infants	11	14	—	6.4 ± 0.5 [5.5, 7.5] months	
RICE	Children	58	78	—	10.8 ± 3.9 [2, 18] years	

**B) Imaging data**					
Cohort name	MRI derivatives		Raw MRI data and EP reconstructions (pre- / post- QC outlier exclusion)		
	MD	Segmentation	GRE - σ, ε_r	EPI - σ	TSE - σ
dHCP[1]	773[2]	873[3] (Tissue labels)	881 / 868	792 / 746	881 / 868
BI-HIE	35	35 (Tissue labels)	—	—	35 / 35
BIBS	—	14 (Whole brain)	14 / 13	—	—
RICE[4,5]	—	78 (Whole brain)	78 / 77	—	—

Supplementary Table S1. Summary of characteristics of the neonatal (dHCP and BI-HIE), infants (BIBS) and children (RICE) cohorts. A) Clinical information relevant to each cohort, including subject and session distribution and age range. For the neonatal cohorts, both gestational age (GA) and post-menstrual age (PMA) are indicated, as well as preterm and term counts. B) Imaging data and MRI derivatives (mean diffusivity — MD and tissue labels) were extracted as part of prior studies.[1–5] Conductivity (σ) and permittivity (ε_r) were reconstructed from three separate MRI series: multi-coil gradient echo (GRE), spin-echo echo planar imaging (EPI) and T2-weighted turbo spin-echo (TSE). After, quality control (QC) outlier exclusion, all three imaging sequences (GRE, EPI and TSE) were simultaneously available in only 725 sessions.

<u>Note on segmentation.</u> For dHCP and BI-HIE cohorts, brain tissue labels were derived from anatomical T2-weighted images using the automated segmentation pipeline of Makropoulos et al., providing nine tissue classes: cortical grey matter, deep grey matter

(dGM), white matter (WM), ventricles, CSF, cerebellum, thalamus and brainstem.[3] BI-HIE tissue labels were manually controlled and corrected. For BIBS and RICE cohorts, where no anatomical images were available, whole-brain masks were delineated manually from body coil magnitude images, encompassing brain parenchyma, CSF and ventricles without tissue-class subdivision.

S2. MRI acquisition parameters

Cohort name	MRI System	Coils	Sequence	Sequence parameters	Initial purpose
dHCP[1]	3T Philips Achieva scanner	Neonatal 32 channel array head coil[6]<br>+<br>Body reference coil	GRE	FA 4°<br>TR 6.2 ms<br>TE 1.56 ms<br>2.6 mm isotropic resolution	SENSE accelerated imaging[7]
			SE-EPI	AP/PA phase encoding directions<br>2.15 mm isotropic resolution	Estimating susceptibility distortions
			T2w-TSE	Sagittal and axial stacks<br>0.8×0.8×1.6 mm^3 resolution<br>0.8 mm slice overlap<br>TR 12000 ms<br>TE 156 ms	Structural analysis
			DTI-MD	4-shell:<br>b = 0, 400, 1000, 2600 $s \cdot mm^{-2}$<br>(20, 64, 88, 128 directions)<br>1.17×1.17×1.5 mm^3 resolution	Diffusion analysis
BI-HIE			TSE	See dHCP	Structural analysis
			MD	See dHCP	Diffusion analysis
BIBS		Adult 32 channel array head coil<br>+<br>Body reference coil	GRE	FA 2°<br>TR 8.9 ms<br>TE 2.3 ms<br>5.5×3.5×5.2 mm^3 resolution	SENSE accelerated imaging[7]
RICE[4,5]			GRE	7.1×5.5×6.0 mm^3 resolution	SENSE accelerated imaging[7]

Supplementary Table S2. MRI acquisition parameters for each cohort. All data were acquired on a single 3T Philips Achieva scanner operating at 128 MHz. Sequence parameters are reported as acquired; none of the sequences were optimised for electrical property tomography. GRE: gradient echo; SE-EPI: spin-echo echo planar imaging; T2w-TSE: T2-weighted turbo spin-echo; DTI-MD: diffusion tensor imaging for mean diffusivity estimation; FA: flip angle; TR: repetition time; TE: echo time; AP/PA: anterior-posterior/posterior-anterior phase encoding directions. The initial purpose column indicates the original research use for which each sequence was acquired. 'See dHCP' indicates identical parameters to the dHCP protocol.

S3. EPT reconstruction pipelines

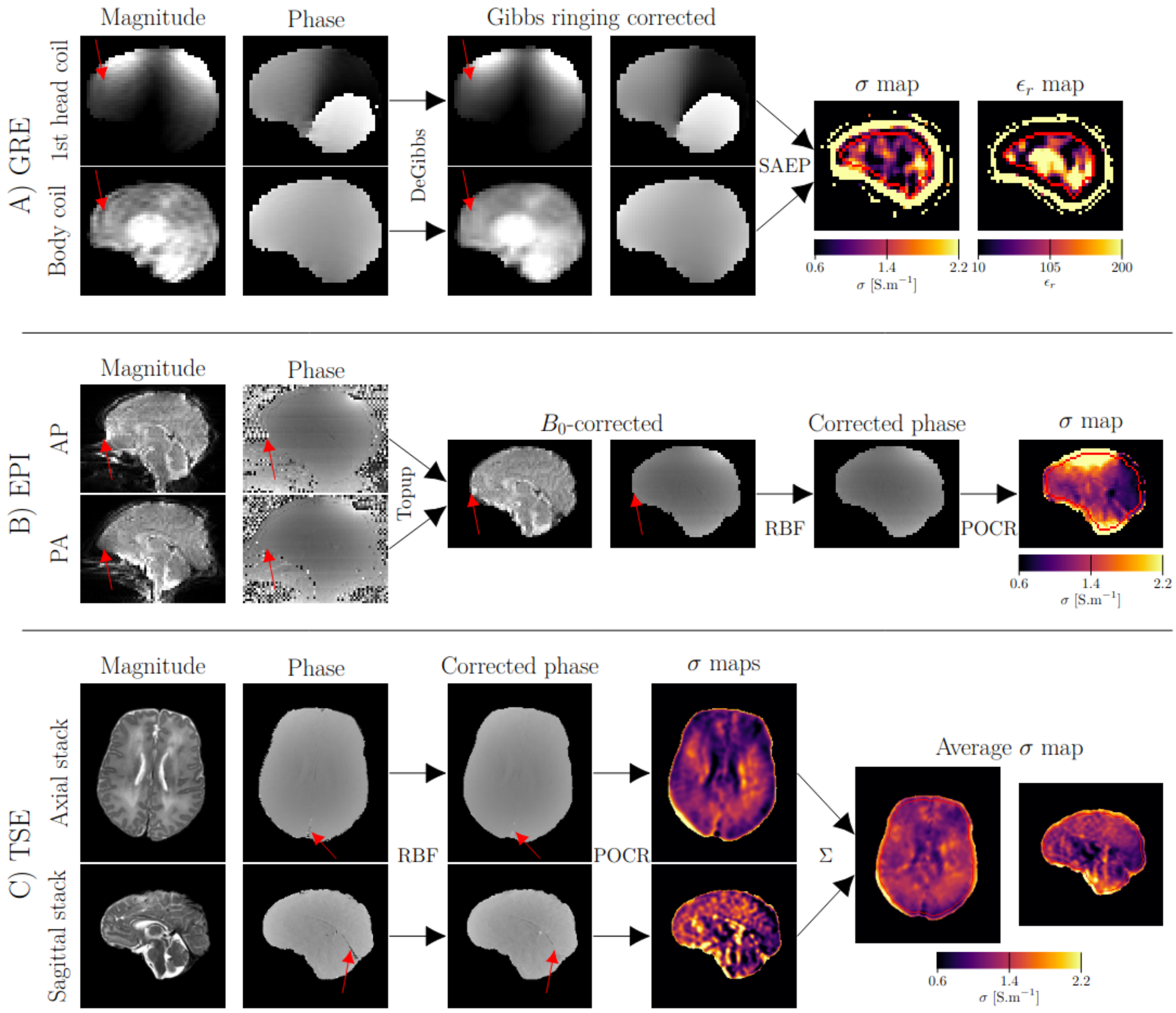

Supplementary Figure S1. Summary of GRE, EPI and TSE pre-processing and EPs reconstruction pipelines. A) Complex multi-channel GRE sensitivities were corrected for ringing artefact prior to conductivity (σ) and permittivity (ε_r) reconstruction using the SAEP method. B) EPI scans acquired with opposite (AP/PA) phase encoding directions were corrected for B_0 inhomogeneity using TopUp algorithm. The phase was corrected for flow void artefact using RBF interpolation prior to σ estimation using the POCR approach. C) σ maps were reconstructed separately from axial and sagittal TSE stacks, then registered and averaged to produce a single σ prediction. Phase images were corrected for blood flow void discontinuities located near the straight sinus as preprocessing. For all pipelines, the median EPs over the eroded brain ROI (red line) were extracted to remove discontinuities at the tissue interface. Red arrows indicate specific artefacts addressed by each preprocessing step.

Common framework

All three pipelines share a common reconstruction framework: electrical property tomography applied to complex MRI data, spatial derivative computation via finite

central difference schemes, and binary erosion of the reconstructed conductivity map to remove boundary artefacts. Pipeline-specific preprocessing addresses sequence-dependent artefacts prior to reconstruction. Permittivity was estimated by the GRE pipeline only and was found to be insufficiently precise for quantitative analysis based on phantom validation (Supplementary Fig. S2); all primary analyses therefore use conductivity only.

GRE

Complex multi-channel GRE images were preprocessed as follows. Gibbs ringing artefacts were removed using the complex-valued 3D sub-voxel shift technique of Kellner et al., implemented in MRtrix.[8,9] Each channel was resampled to the acquired resolution via *k*-space truncation, Gibbs-corrected, and smoothed with an isotropic 3D Gaussian filter (STD kernel = 1.5 × image resolution: dHCP 3.9 mm, BIBS 5.25 mm, RICE 8.25 mm). Channel dimensionality was reduced from 32 to 12 using singular value decomposition (SVD), retaining the dominant components to reduce noise in relative sensitivities and computational load.

Conductivity and permittivity were reconstructed within the brain ROI using the SAEP method based on the homogeneous Helmholtz equation, with spatial derivatives computed by central difference schemes and the inverse problem solved by Moore-Penrose inversion at each voxel.[10] Reconstructed maps were eroded to remove boundary oscillations (dHCP 15.6 mm, BIBS 24.5 mm, RICE 33.0 mm radius), with the amount depending on spatial resolution. See Supplementary Fig. S1A.

EPI

EPI images acquired with opposing phase encoding directions (AP/PA) were corrected for B0 inhomogeneity using the FSL TOPUP algorithm.[11,12] Where multiple AP/PA pairs were available, the pair with the lowest z-smoothness score, defined as the standard deviation of slice-to-slice signal difference in the *z*-direction, was selected to minimise motion contamination. Geometric correction was applied separately to the real and imaginary components before recombination and phase extraction.

Phase discontinuities near the straight sinus arising from flow void artefact were corrected slice-by-slice using radial basis function (RBF) thin-plate spline interpolation. Phase images were smoothed with a 2D slice-by-slice Gaussian filter (STD = 2.1 mm).

Conductivity was reconstructed using the POCR method based on the heterogeneous Helmholtz equation, solved as a sparse linear system using the generalised minimal residual (GMRES) iterative method with diffusion regularisation constant $c = 0.05$.[13] Due to slice-to-slice motion, spatial derivatives and the Laplacian were computed in the x-y plane only, with 3D regularisation applied to maintain spatial cohesion across slices. The resulting conductivity map was multiplied by a factor of 1.5 to compensate for the

missing through-plane derivative, under the assumption of local isotropy, an approximation that introduces spatially variable error and represents a known limitation of this pipeline.[13,14] Maps were eroded by 8.4 mm to remove boundary artefacts. See Supplementary Fig. S1B.

TSE

TSE data from the dHCP and BI-HIE cohorts were acquired as separate axial and sagittal stacks. Complex multi-shot stacks were motion-corrected during MR reconstruction using a *k*-space-based approach.[1] Where multiple stacks per orientation were available, the stack with the lowest *z*-smoothness score was selected. Flow void phase discontinuities near the straight sinus were corrected slice-by-slice using thin-plate spline interpolation. Phase images were smoothed with a 2D slice-by-slice Gaussian filter (STD = 1.6 mm).

Conductivity was reconstructed independently from axial and sagittal stacks using a hybrid 2D/3D POCR implementation: 2D spatial derivatives to mitigate slice-to-slice motion, with 3D regularisation (c = 0.05) to ensure spatial consistency.[13] The two reconstructed maps were registered and averaged to produce a single conductivity estimate per subject, partially compensating for the through-plane derivative limitation inherent to 2D reconstruction. The final map was eroded by 4.8 mm. See Supplementary Fig. S1C.

S4. Statistical model specification and diagnostics

All linear mixed-effects (LME) models were fitted using maximum likelihood (ML) rather than restricted maximum likelihood (REML) to permit likelihood ratio testing of nested fixed effects models. Subject-wise random intercepts were included throughout; random slopes were not estimated as the maximum of two sessions per subject precluded robust slope estimation.

<u>Multicollinearity</u>. Variance inflation factors (VIF) were computed for all multivariate models prior to fitting. All VIF values fell below the conventional threshold of 5, indicating acceptable multicollinearity and reliable coefficient estimation.

<u>Model selection</u>. Interaction terms between predictors, including PMA×PTB and polynomial terms (PMA^2, PMA^3), were tested but did not improve model fit by likelihood ratio test and were excluded from final models.

<u>Heteroscedasticity</u>. Standard LME models assume homoscedastic residuals. To assess whether heteroscedasticity materially affected fixed effect estimates, LME results were compared against linear models incorporating HC3 heteroscedasticity-consistent covariance matrix estimation. Coefficient estimates and standard errors were consistent across both approaches, indicating that heteroscedasticity did not substantially bias the reported fixed effect estimates or their associated standard errors.

Outlier and leverage diagnostics**.** LME results were compared against robust linear models fitted to the same data, coefficient estimates were consistent across both approaches, indicating that outliers did not materially influence model parameters. Leverage point sensitivity was assessed by refitting models after excluding observations outside the 1st–99th percentile of each predictor's range; results were unchanged.

Study design

Blinding and randomisation. Quality control exclusions were based on objective quantitative criteria applied identically across all sessions without reference to clinical group. This was a retrospective observational study; randomisation was not applicable.

Sample size. No a priori power calculation was performed as this was a retrospective observational study.

S5. Implementation details

Implementation of the SAEP and POCR EPT reconstruction methods was carried out in Python (3.10) using standard medical imaging and scientific libraries: NiBabel (5.1.0), NumPy (1.26.4) and SciPy (1.10). Image rigid registration transformations were applied through MIRTK toolkit (2.0). Low resolution GRE images were corrected for Gibbs ringing via MRtrix software (3.0.4) while dHCP EPI images were corrected for B_0 distortion using FSL toolbox (6.0.7.2). Data visualization and statistical analysis was carried out in Python using statsmodels (0.14.2) and seaborn (0.10.1) packages. Finally, we used ITK-SNAP (3.6.0) to visualize three-dimensional medical images and perform manual segmentation. Experiments were performed using an Intel Xeon Silver 4214R CPU (2.40 GHz, 24 cores) and 96 GB RAM.

Supplementary results

S6. GRE phantom validation

To assess EPT reconstruction accuracy, three saline phantoms of varying concentration (3, 6 and 9 g·L^{-1}) were scanned on a 3T Philips Achieva scanner using the same multi-coil GRE sequence as the neonatal data: complex low-resolution (2.6 mm isotropic) sensitivity reference scans with 32 head coil channels and one body coil reference. The identical GRE processing pipeline was applied. Brain masks were defined by Otsu thresholding. Ground truth conductivity and permittivity were measured using a dielectric probe. Median and spatial standard deviation were extracted over the eroded ROI as summary measures.

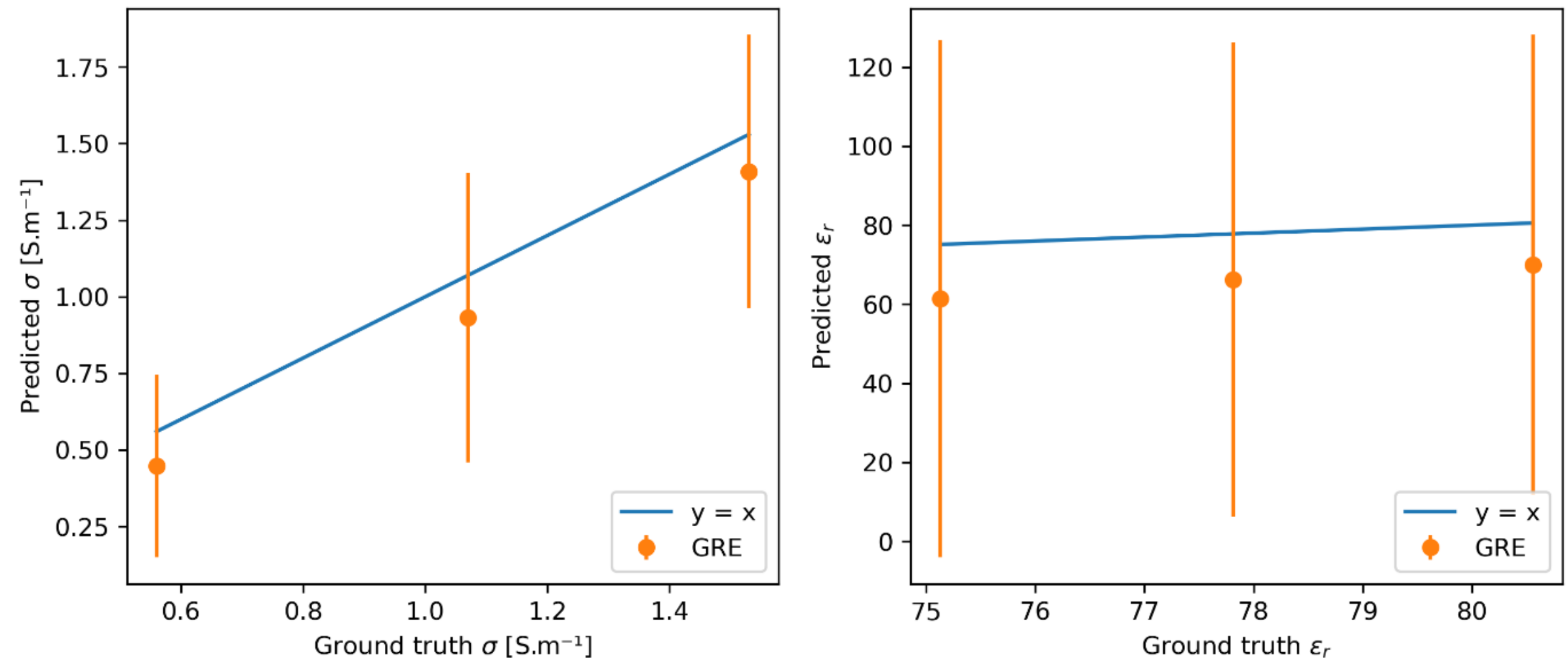

Supplementary Figure S2. Comparison of ground truth and predicted conductivity (σ) and permittivity (ε_r) measurements on 3T GRE spherical phantoms with varying saline concentrations. Ground truth values were measured using a dielectric probe. Median predicted measurements are reported along with their associated standard deviations (STDs).

Analysis of Supplementary Fig. S2. Conductivity showed reasonable agreement with ground truth values across all three phantoms, with EPT-based estimates within 8–20% of probe measurements. Spatial standard deviations ranged from 29–53% of the mean, reflecting the noise amplification inherent to the third-order spatial derivatives required by SAEP reconstruction. Permittivity estimates were within 13–18% of ground truth means but showed spatial standard deviations of 72–87%, much larger than for conductivity. This variance reflects a fundamental signal limitation: the non-optimised GRE sequence provides insufficient B1 information for reliable permittivity estimation. Permittivity is therefore excluded from all primary analyses in this study. Permittivity results are presented in Supplementary Methods S11 for completeness.

S7. Cross-method agreement and robustness

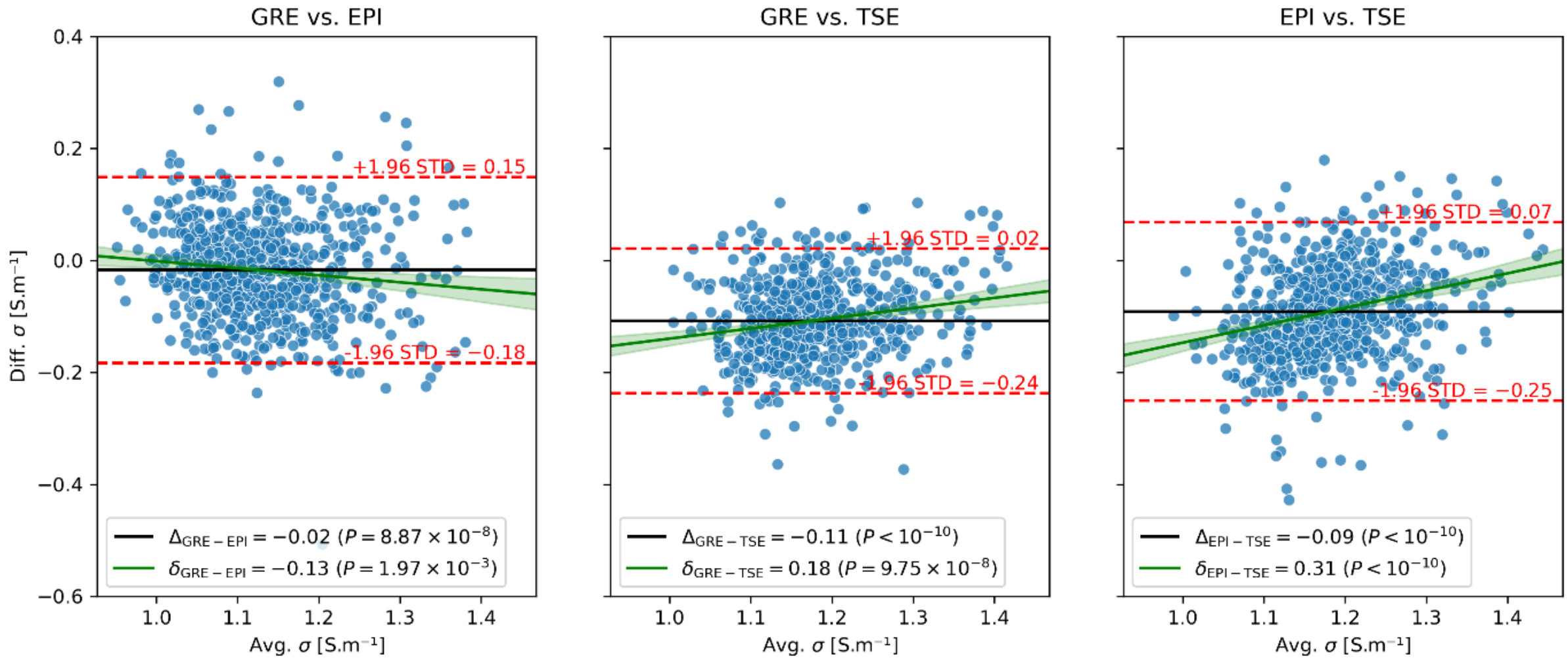

Supplementary Figure S3. Bland-Altman agreement among neonatal GRE, EPI and TSE brain conductivity (σ) measurements (*n* = 725 sessions with all three methods available). For each pairwise comparison, the difference between methods is plotted against their mean. The black horizontal line indicates the mean difference (systematic bias, Δ); red dashed lines indicate limits of agreement. The green line shows the regression of difference on mean, with slope δ quantifying proportional bias. All systematic and proportional biases were statistically significant.

Analysis of Supplementary Fig. S3. GRE and EPI showed close absolute agreement, while TSE measurements were systematically higher than both. All pairs showed significant proportional biases, indicating that method disagreement increases at higher conductivity values, predominantly in the most preterm neonates.

**A) Full linear mixed-effects model (R_m^2 = 0.54)**<br>σ ~ Type + Type × PMA + PMA + (1 | Subject)				
	Coef.	Std. Err.	$P > \|z\|$	95% CI
Type[GRE]	1.115	0.002	$P < 10^{-10}$	[1.110, 1.120]
Type[EPI]	1.132	0.002	$P < 10^{-10}$	[1.127, 1.137]
Type[TSE]	1.223	0.002	$P < 10^{-10}$	[1.219, 1.228]
Type[GRE]:PMA	-0.015	0.001	$P < 10^{-10}$	[-0.017, -0.014]
Type[EPI]:PMA	-0.015	0.001	$P < 10^{-10}$	[-0.016, -0.014]
Type[TSE]:PMA	-0.013	0.001	$P < 10^{-10}$	[-0.015, -0.012]
**B) Restricted linear mixed-effects model (R_m^2 = 0.54)**<br>σ ~ Type + PMA + (1 | Subject)				
	Coef.	Std. Err.	$P > \|z\|$	95% CI
Type[GRE]	1.115	0.002	$P < 10^{-10}$	[1.110, 1.120]
Type[EPI]	1.132	0.002	$P < 10^{-10}$	[1.127, 1.137]
Type[TSE]	1.223	0.002	$P < 10^{-10}$	[1.218, 1.228]
PMA	-0.015	0.001	$P < 10^{-10}$	[-0.016, -0.014]

Supplementary Table S3. Summaries of the univariate linear mixed-effects (LME) models fitted to the GRE, EPI, TSE conductivity (*σ*) values (*n* = 725) with post-menstrual age (PMA) as explanatory variable. The full model A) includes intercept and PMA-linear effect interaction terms with respect to GRE, EPI, TSE measurement types, while the restricted model B) includes only intercept interactions. Estimated coefficients are reported along with their associated standard error, significance level and 95% confidence interval. The marginal coefficients of determination R_m^2 are also indicated.

Analysis of Supplementary Table S3. A likelihood ratio test detected a statistically significant but negligible interaction between measurement type and PMA slope ($\chi^2(2)$ = 7.22, P = 0.027). However, PMA slope estimates were identical across GRE and EPI (−0.015 $S \cdot m^{-1} \cdot week^{-1}$) and marginally shallower for TSE (−0.013 $S \cdot m^{-1} \cdot week^{-1}$) with overlapping confidence intervals across all three methods. Marginal R_m^2 was identical between the full and restricted models (0.54), confirming that removing the interaction terms did not reduce explanatory power. The restricted model with a common PMA slope was therefore adopted for subsequent analyses.

S8. Detailed statistical models - Developmental

Brain conductivity ~ MD + RVV + PMA

**Multivariate linear mixed-effects model (R_m^2 = 0.59)**<br>σ ~ MD + RVV + PMA + (1 | Subject)				
	Coef.	Std. Err.	$P > \|z\|$	95% CI
Intercept	1.174	0.002	$P < 10^{-10}$	[1.170, 1.177]
MD	0.365	0.045	$P < 10^{-10}$	[0.277, 0.452]
RVV	0.017	0.004	$P = 4.90\times10^{-5}$	[0.009, 0.025]
PMA	-0.007	0.001	$P < 10^{-10}$	[-0.008, -0.005]

Supplementary Table S4. Summary of the multivariate linear mixed-effects (mvLME) model fitted to neonatal brain conductivity (σ) values (*n* = 662) with mean diffusivity (MD), relative ventricle volume (RVV) and post-menstrual age (PMA) as predictor variables. Estimated coefficients are reported along with their associated standard error, significance level and 95% confidence interval. The marginal coefficient of determination R_m^2 is also indicated. Unstandardised coefficients are reported here; standardised equivalents are shown in Fig. 3B.

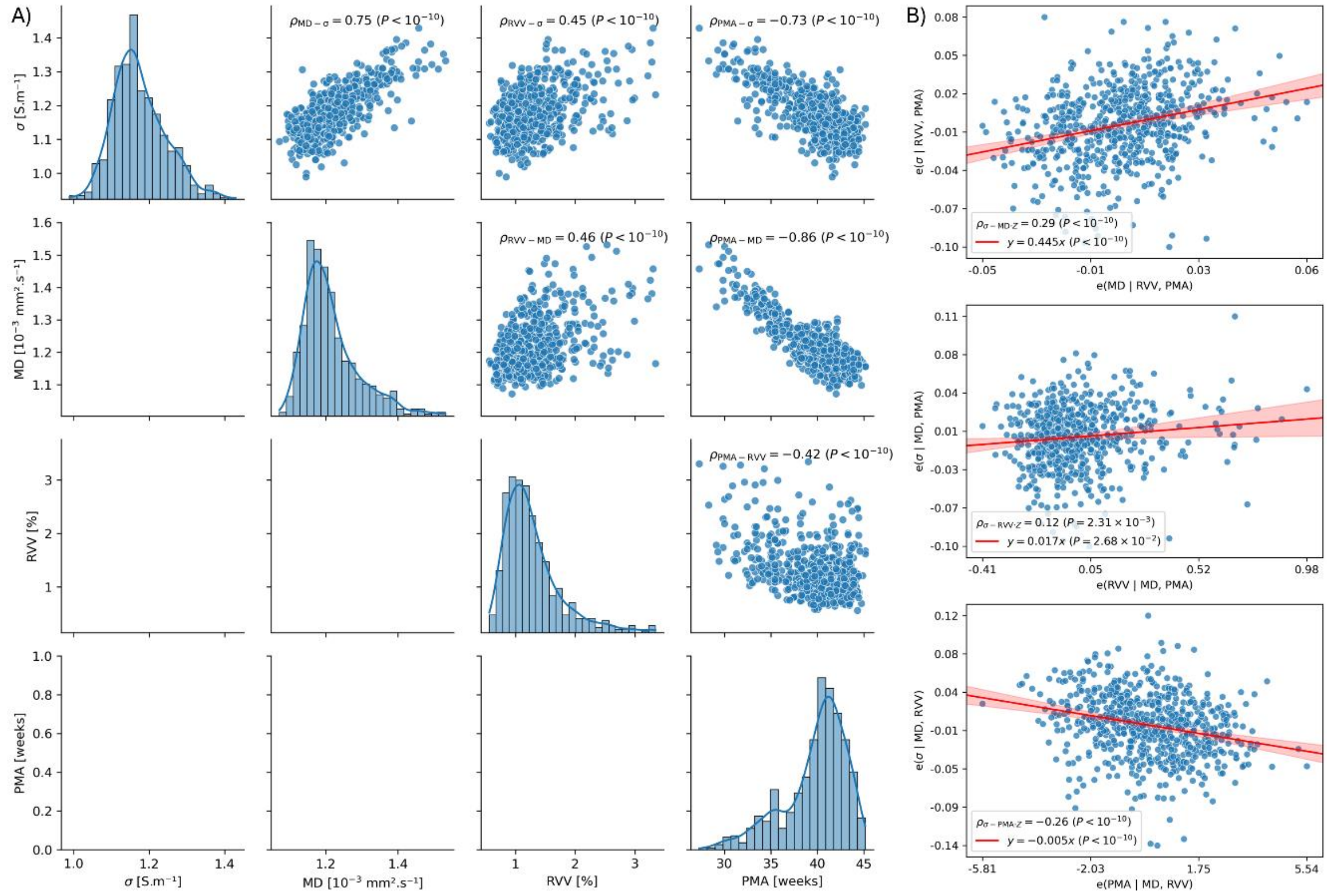

Supplementary Figure S4. Association between brain conductivity (σ), mean diffusivity (MD), relative ventricle volume (RVV) and post-menstrual age (PMA) in neonatal cohort (*n* = 662). A) Pairwise relationships between the variables of interest. Pearson's correlation coefficients ρ_{XY} are reported with their associated significance level. B) Partial regression plots between brain σ and PMA (MD or RVV, respectively), controlling for the other two variables. Partial correlation coefficients $\rho_{XY\cdot\mathbf{Z}}$ are computed using the residuals $e(X|\mathbf{Z})$ and $e(Y|\mathbf{Z})$ and reported with their associated significance level.

Analysis of Supplementary Fig. S4. A) There are strong pairwise correlations between σ, MD, RVV and PMA. The σ-MD correlation is consistent with the known relationship between conductivity and tissue water content. Both σ and MD are negatively associated with PMA, reflecting progressive water reduction during brain maturation, with MD showing a stronger developmental association than σ, likely reflecting greater measurement noise in EPT-derived conductivity. RVV decreases with PMA as brain tissue volume increases relative to intracranial volume, and its mild association with σ reflects partial volume contamination from CSF, whose conductivity substantially exceeds that of brain parenchyma. MD and RVV were therefore included as covariates in multivariate models (Supplementary Table S4) to isolate the independent contribution of PMA to conductivity. B) All three predictors retained significant independent associations with σ after mutual adjustment, though partial correlations were substantially attenuated relative to marginal values, reflecting the high intercorrelations between predictors.

Brain conductivity ~ MD + RVV + PMA + PTB

**Multivariate linear mixed-effects model (R_m^2 = 0.35)**<br>σ ~ MD + RVV + PMA + PTB + (1 | Subject)				
	Coef.	Std. Err.	$P > \|z\|$	95% CI
Intercept	1.153	0.001	$P < 10^{-10}$	[1.151, 1.155]
MD	0.323	0.055	$P = 4.29 \times 10^{-9}$	[0.215, 0.431]
RVV	0.017	0.005	$P = 0.001$	[0.007, 0.027]
PTB	-0.002	0.001	$P = 8.42 \times 10^{-5}$	[0.001, 0.003]
PMA	-0.008	0.001	$P < 10^{-10}$	[-0.010, -0.006]

Supplementary Table S5. Summary of the multivariate linear mixed-effects (mvLME) model fitted to neonatal brain conductivity (σ) values (*n* = 534) with mean diffusivity (MD), relative ventricle volume (RVV), preterm birth (PTB) and post-menstrual age (PMA) as predictor variables. Estimated coefficients are reported along with their associated standard error, significance level and 95% confidence interval. The marginal coefficient of determination R_m^2 is also indicated. Unstandardised coefficients are reported here; standardised equivalents are shown in Fig. 4A.

Tissue conductivity ~ MD + RVV + PMA + PTB

**Multivariate linear mixed-effects linear model (R_m^2 = 0.31)**<br>σ ~ Tissue × (MD + RVV + PTB + PMA) + (1 | Subject)				
	Coef.	Std. Err.	$P > \|z\|$	95% CI
Tissue[dGM]	1.131	0.002	$P < 10^{-10}$	[1.127, 1.136]
Tissue[WM]	1.141	0.002	$P < 10^{-10}$	[1.136, 1.145]
Tissue[dGM]:MD	0.302	0.067	$P = 6.15\times10^{-6}$	[0.171, 0.434]
Tissue[WM]:MD	0.242	0.034	$P < 10^{-10}$	[0.176, 0.308]
Tissue[dGM]:RVV	0.035	0.006	$P = 3.97\times10^{-8}$	[0.023, 0.048]
Tissue[WM]:RVV	0.012	0.006	$P = 0.064$	[-0.001, 0.025]
Tissue[dGM]:PTB	0.003	0.001	$P = 9.54\times10^{-8}$	[0.002, 0.005]
Tissue[WM]:PTB	0.001	0.001	$P = 0.067$	[0.000, 0.003]
Tissue[dGM]:PMA	-0.011	0.002	$P = 1.13\times10^{-10}$	[-0.014, -0.007]
Tissue[WM]:PMA	-0.008	0.001	$P = 2.89\times10^{-9}$	[-0.011, -0.005]
	Diff.	Std. Err.	$P > \|z\|$	95% CI
Tissue×PTB	0.002	0.001	$P = 0.002$	[0.001, 0.003]

Supplementary Table S6. Summary of the multivariate linear mixed-effects (mvLME) model fitted to the neonatal deep grey matter (dGM) and white matter (WM) conductivity (σ) values (*n* = 534) with mean diffusivity (MD), relative ventricle volume (RVV), preterm birth (PTB) and post-menstrual age (PMA) as explanatory variables. The model includes interaction terms with respect to dGM and WM tissue types. Estimated coefficients are reported along with their associated standard error, significance level and 95% confidence interval. The marginal coefficient of determination R_m^2 is also indicated. Unstandardised coefficients are reported here; standardised equivalents are shown in Fig. 4B.

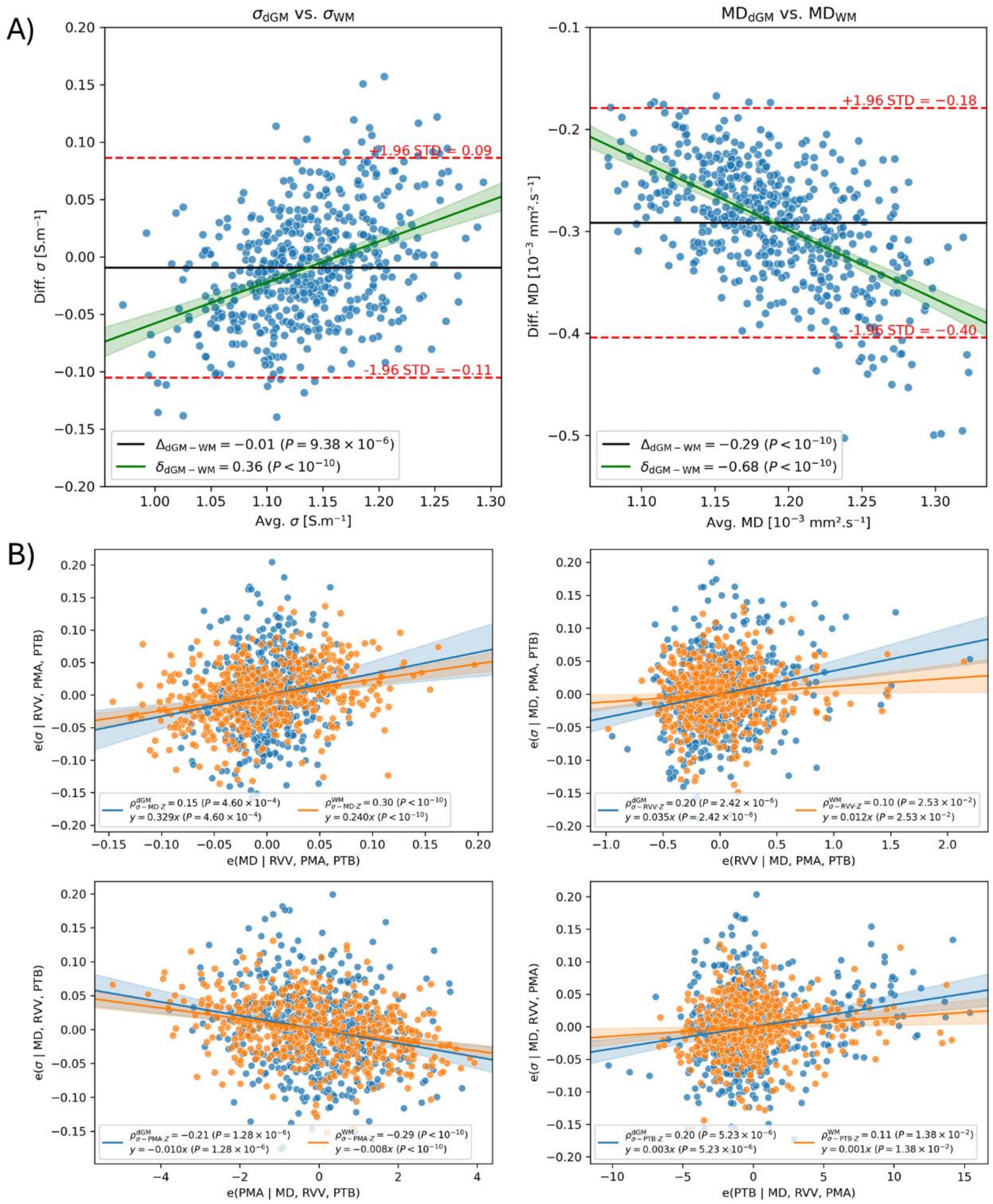

Supplementary Figure S5. Association between conductivity (σ), mean diffusivity (MD), relative ventricle volume (RVV), preterm birth (PTB) and post-menstrual age (PMA) within deep grey matter (dGM) and white matter (WM) tissues in neonatal cohort (*n* = 534). A) Bland-Altman plot between tissue-level σ and MD measurements. Systematic (Δ) and proportional (δ) biases are indicated with their significance levels. B) Partial regression plots between brain σ and PMA (MD, RVV, or PTB respectively), controlling for the other three variables. Partial correlation coefficients $\rho_{XY \cdot \mathbf{Z}}$ are computed using the residuals $e(X|\mathbf{Z})$ and $e(Y|\mathbf{Z})$ and reported with their associated significance level.

Analysis of Supplementary Fig. S5. A). Bland-Altman analysis revealed a small but significant systematic bias between dGM and WM conductivity, with WM marginally more conductive than dGM. This contrasts with adult reference values where GM typically exceeds WM conductivity (IT'IS database), likely reflecting incomplete tissue differentiation in the neonatal brain and residual partial volume effects from spatial smoothing. A significant positive proportional bias suggests increasing tissue conductivity contrast with advancing maturation. For MD, a larger systematic offset and strong negative proportional bias confirm that, in our study, MD distinguishes tissue types more effectively than conductivity at this developmental stage. B) The persistence of significant PMA and PTB partial associations after controlling for MD and RVV confirms that developmental and prematurity-related conductivity changes are not fully explained by water content or ventricular volume.

S9. Detailed statistical models - HIE

Tissue conductivity ~ PMA + HIE & Tissue MD ~ PMA + MD

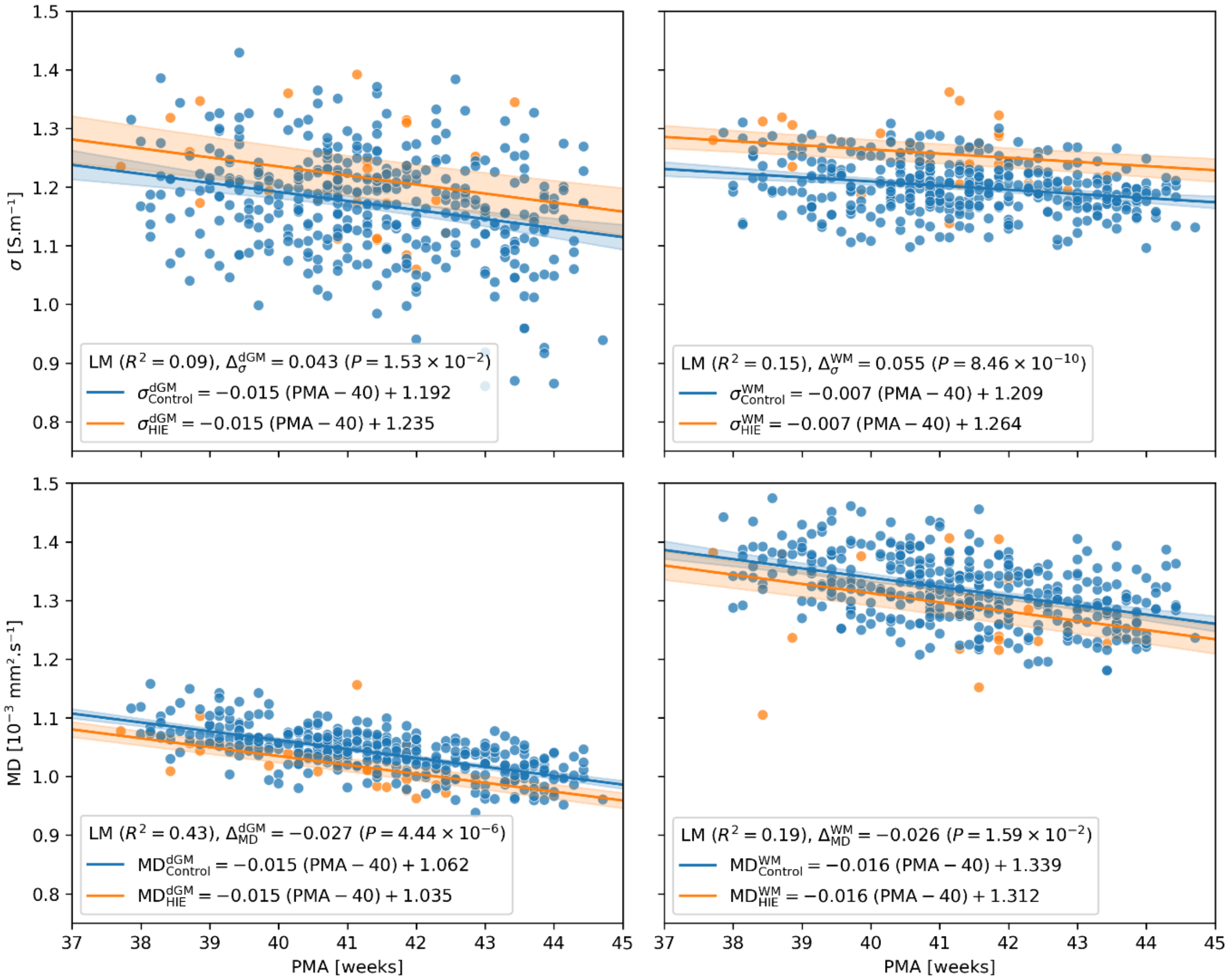

Supplementary Figure S6. Conductivity (σ) and mean diffusivity (MD) with respect to post-menstrual age (PMA) within deep grey matter (dGM) and white matter (WM) tissues of the control dHCP (*n* = 379) and BI-HIE (*n* = 25) cohorts. For each metric and within each tissue, a linear model (LM) was fitted to all control and HIE values with PMA as exploratory variable. The coefficient of determination R^2, 95% confidence interval and group mean difference (Δ) are reported for each model. Tissue-level group mean differences are summarised in Fig. 5B.

Tissue conductivity ~ MD + RVV + PMA + HIE

**Multivariate linear mixed-effects linear model (R_m^2 = 0.19)**<br>σ ~ Tissue × (MD + RVV + PMA + HIE) + (1 | Subject)				
	Coef.	Std. Err.	*P* > |*z*|	95% CI
Tissue[dGM]	1.171	0.003	$P < 10^{-10}$	[1.164, 1.178]
Tissue[WM]	1.200	0.003	$P < 10^{-10}$	[1.193, 1.206]
Tissue[dGM]:Type[HIE]	0.050	0.014	$P = 5.44\times10^{-4}$	[0.022, 0.078]
Tissue[WM]:Type[HIE]	0.062	0.014	$P = 1.50\times10^{-5}$	[0.034, 0.090]
Tissue[dGM]:MD	0.605	0.116	$P = 1.94\times10^{-7}$	[0.377, 0.832]
Tissue[WM]:MD	0.256	0.062	$P = 4.20\times10^{-5}$	[0.133, 0.378]
Tissue[dGM]:RVV	0.036	0.014	$P = 0.021$	[0.008, 0.064]
Tissue[WM]:RVV	-0.001	0.014	$P = 0.951$	[-0.029, 0.027]
Tissue[dGM]:PMA	-0.006	0.003	$P = 0.178$	[-0.012, -0.001]
Tissue[WM]:PMA	-0.003	0.002	$P = 1.50\times10^{-5}$	[-0.008, 0.001]

Supplementary Table S7: Summary of the multivariate linear mixed-effects (mvLME) model fitted to the neonatal deep grey matter (dGM) and white matter (WM) conductivity (σ) values in control dHCP (*n* = 379) and BI-HIE (*n* = 25) cohorts. Mean diffusivity (MD), relative ventricle volume (RVV) and post-menstrual age (PMA) are included as explanatory variables. The model includes interaction terms with respect to dGM and WM tissue types as well as cohort status (i.e. control and HIE). Estimated coefficients are reported along with their associated standard error, significance level and 95% confidence interval. The marginal coefficient of determination R_m^2 is also indicated. Unstandardised coefficients are reported here.

S10. Exploratory multivariate HIE classification

A multivariate logistic binary classifier model was fitted to predict HIE/control status using tissue-level quantitative imaging measurements (i.e., σ_{dGM}, σ_{WM}, MD_{dGM}, MD_{WM}) and subject-level variables (i.e., RVV and PMA) as predictors. PMA was included as a control variable to account for potential cohort-specific differences in scan timing. Variables were *z*-score normalized and standardized odds ratios (OR) were computed from the estimated model coefficients. Odds ratios were reported along their confidence intervals. Model goodness of fit was measured using McFadden coefficient of determination (R^2_{McF}).

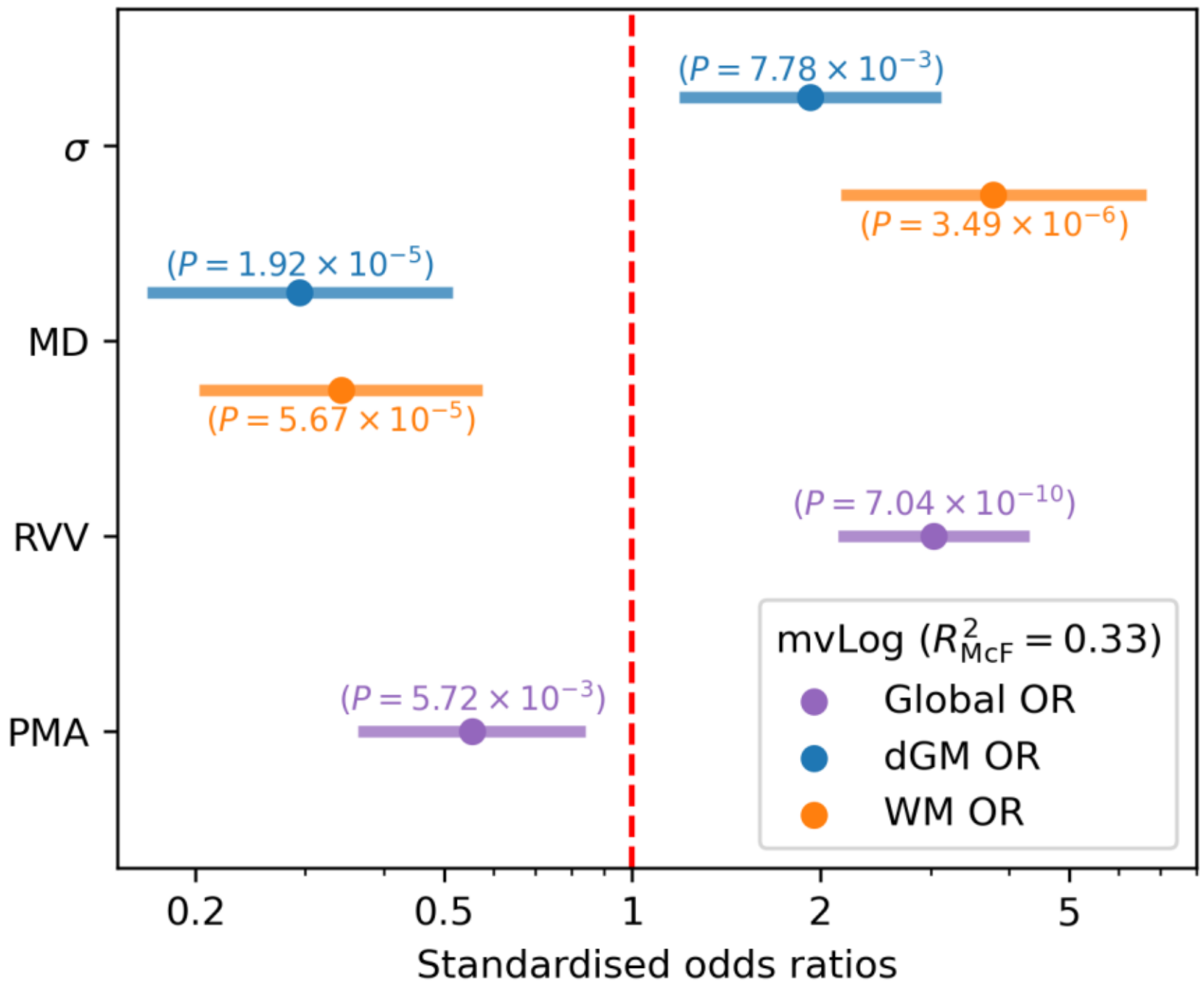

Supplementary Figure S7. Brain injury classification model using imaging and clinical predictors fitted on control (n = 379) and HIE (n = 25) neonatal data. Odds ratios (OR) of post-menstrual age (PMA), relative ventricle volume (RVV) and tissue mean diffusivity (MD) and conductivity (σ) for HIE classification. Odds ratios were computed using a multivariate logistic model (mvLog), with McFadden coefficient of determination (R^2_{McF}) indicated. Effects are reported along with their 95% confidence intervals and exact *P*-values are shown.

<u>Analysis of Supplementary Fig. S7.</u> Higher conductivity was strongly associated with increased odds of HIE, with a substantially larger effect in WM than in. In contrast, higher MD was associated with reduced odds of HIE in both tissues, with comparable effect sizes across tissue types. RVV was independently associated with an increased odds of HIE, indicating structural feature discrimination. Finally, PMA showed a modest contribution, which is likely to reflect cohort-specific differences in scan timing distribution rather than a direct age effect.

S11. Permittivity analysis

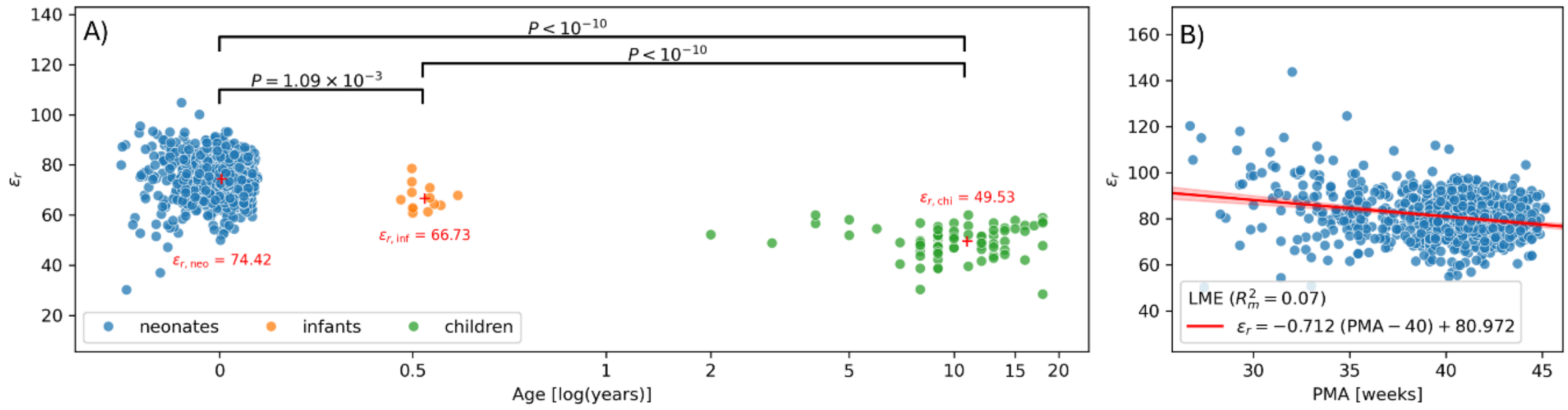

Supplementary Figure S8. Brain electrical permittivity (ε_r) of neonates, infants and children cohorts. A) Scatter plot of ε_r GRE estimates in neonates (26 to 45 weeks post-menstrual age – PMA, *n* = 868), infants (~ 6 months old, *n* = 13) and children (2 to 18 years old, *n* = 77) versus age. The scale is linear up to one, then logarithmic. Age 0 is defined as 40 weeks PMA. Group means were compared using a mixed-effects model and Bonferroni correction was applied for multiple comparison. Group means are reported for each cohort. B) Linear regression of neonatal GRE ε_r measurements (*n* = 868) against PMA. Regressions were performed using linear mixed-effects (LME) models. The marginal coefficient of determination (R_m^2) and the 95% confidence interval are indicated.

Supplementary references

1. Edwards AD, Rueckert D, Smith SM, et al. The Developing Human Connectome Project Neonatal Data Release. *Front Neurosci*. 2022;16. doi:10.3389/fnins.2022.886772

2. Gale-Grant O, Fenn-Moltu S, França LGS, et al. Effects of gestational age at birth on perinatal structural brain development in healthy term-born babies. *Hum Brain Mapp*. 2022;43(5):1577-1589. doi:10.1002/hbm.25743

3. Makropoulos A, Robinson EC, Schuh A, et al. The developing human connectome project: A minimal processing pipeline for neonatal cortical surface reconstruction. *Neuroimage*. 2018;173:88-112. doi:10.1016/j.neuroimage.2018.01.054

4. Vecchiato K, Egloff A, Carney O, et al. Evaluation of DISORDER: Retrospective Image Motion Correction for Volumetric Brain MRI in a Pediatric Setting. *AJNR Am J Neuroradiol*. 2021;42(4):774-781. doi:10.3174/ajnr.A7001

5. Casella C, Vecchiato K, Cromb D, et al. Widespread, depth-dependent cortical microstructure alterations in pediatric focal epilepsy. *Epilepsia*. 2024;65(3):739-752. doi:10.1111/epi.17861

6. Hughes EJ, Winchman T, Padormo F, et al. A dedicated neonatal brain imaging system. *Magn Reson Med*. 2017;78(2):794-804. doi:10.1002/mrm.26462

7. Pruessmann KP, Weiger M, Scheidegger MB, Boesiger P. SENSE: sensitivity encoding for fast MRI. *Magn Reson Med*. 1999;42(5):952-962.

8. Tournier JD, Smith R, Raffelt D, et al. MRtrix3: A fast, flexible and open software framework for medical image processing and visualisation. *Neuroimage*. 2019;202:116137. doi:10.1016/j.neuroimage.2019.116137

9. Kellner E, Dhital B, Kiselev VG, Reisert M. Gibbs-ringing artifact removal based on local subvoxel-shifts. *Magn Reson Med*. 2016;76(5):1574-1581. doi:10.1002/mrm.26054

10. Marques JP, Sodickson DK, Ipek O, Collins CM, Gruetter R. Single acquisition electrical property mapping based on relative coil sensitivities: A proof-of-concept demonstration. *Magn Reson Med*. 2015;74(1):185-195. doi:https://doi.org/10.1002/mrm.25399

11. Andersson JLR, Skare S, Ashburner J. How to correct susceptibility distortions in spin-echo echo-planar images: Application to diffusion tensor imaging. *Neuroimage*. 2003;20(2):870-888. doi:10.1016/S1053-8119(03)00336-7

12. Jenkinson M, Beckmann CF, Behrens TEJ, Woolrich MW, Smith SM. FSL. *Neuroimage*. 2012;62(2):782-790. doi:10.1016/j.neuroimage.2011.09.015

13. Gurler N, Ider YZ. Gradient-based electrical conductivity imaging using MR phase. *Magn Reson Med*. 2017;77(1):137-150. doi:10.1002/mrm.26097

14. Mandija S, Sbrizzi A, Katscher U, Luijten PR, van den Berg CAT. Error analysis of helmholtz-based MR-electrical properties tomography. *Magn Reson Med*. 2018;80(1):90-100. doi:10.1002/mrm.27004
